# Knockout of secretin ameliorates biliary and liver phenotypes during alcohol-induced hepatotoxicity

**DOI:** 10.1186/s13578-022-00945-w

**Published:** 2023-01-09

**Authors:** Konstantina Kyritsi, Nan Wu, Tianhao Zhou, Guido Carpino, Leonardo Baiocchi, Lindsey Kennedy, Lixian Chen, Ludovica Ceci, Alison Ann Meyer, Nipuni Barupala, Antonio Franchitto, Paolo Onori, Burcin Ekser, Eugenio Gaudio, Chaodong Wu, Corinn Marakovits, Sanjukta Chakraborty, Heather Francis, Shannon Glaser, Gianfranco Alpini

**Affiliations:** 1 Division of Gastroenterology and Hepatology, Department of Medicine, Indiana University School of Medicine, Indianapolis, IN USA; 2 Department of Anatomical, Histological, Forensic Medicine and Orthopedics Sciences, La Sapienza University of Rome, Rome, Italy; 3 Unit of Hepatology, Tor Vergata University, Rome, Italy; 4 Division of Research, Indiana Center for Liver Research, Gastroenterology, Medicine, Richard L. Roudebush VA Medical Center and Indiana University, 702 Rotary Circle, Rm. 013C, Indianapolis, IN 46202-2859 USA; 5 Department of Movement, Human and Health Sciences, University of Rome “Foro Italico”, Rome, Italy; 6 Division of Transplant Surgery, Department of Surgery, Indiana University, Indianapolis, IN USA; 7 Department of Nutrition, Texas A&M University, College Station, TX USA; 8 Department of Medical Physiology, Texas A&M University School of Medicine, 8447 Riverside Parkway, MREB II, Room 2342, Bryan, TX 77807-3260 USA

**Keywords:** Biliary senescence, Ductular reaction, Fatty liver diseases, Hepatic steatosis, Lipogenesis

## Abstract

**Background:**

Alcohol-related liver disease (ALD) is characterized by ductular reaction (DR), liver inflammation, steatosis, fibrosis, and cirrhosis. The secretin (Sct)/secretin receptor (SR) axis (expressed only by cholangiocytes) regulates liver phenotypes in cholestasis. We evaluated the role of Sct signaling on ALD phenotypes.

**Methods:**

We used male wild-type and Sct^−/−^ mice fed a control diet (CD) or ethanol (EtOH) for 8 wk. Changes in liver phenotypes were measured in mice, female/male healthy controls, and patients with alcoholic cirrhosis. Since Cyp4a10 and Cyp4a11/22 regulate EtOH liver metabolism, we measured their expression in mouse/human liver. We evaluated: (i) the immunoreactivity of the lipogenesis enzyme elongation of very-long-chain fatty acids 1 (Elovl, mainly expressed by hepatocytes) in mouse/human liver sections by immunostaining; (ii) the expression of miR-125b (that is downregulated in cholestasis by Sct) in mouse liver by *q*PCR; and (iii) total bile acid (BA) levels in mouse liver by enzymatic assay, and the mRNA expression of genes regulating BA synthesis (cholesterol 7a-hydroxylase, Cyp27a1, 12a-hydroxylase, Cyp8b1, and oxysterol 7a-hydroxylase, Cyp7b11) and transport (bile salt export pump, Bsep, Na^+^-taurocholate cotransporting polypeptide, NTCP, and the organic solute transporter alpha (OSTa) in mouse liver by *q*PCR.

**Results:**

In EtOH-fed WT mice there was increased biliary and liver damage compared to control mice, but decreased miR-125b expression, phenotypes that were blunted in EtOH-fed Sct^−/−^ mice. The expression of Cyp4a10 increased in cholangiocytes and hepatocytes from EtOH-fed WT compared to control mice but decreased in EtOH-fed Sct^−/−^ mice. There was increased immunoreactivity of Cyp4a11/22 in patients with alcoholic cirrhosis compared to controls. The expression of miR-125b decreased in EtOH-fed WT mice but returned at normal values in EtOH-fed Sct^−/−^ mice. Elovl1 immunoreactivity increased in patients with alcoholic cirrhosis compared to controls. There was no difference in BA levels between WT mice fed CD or EtOH; BA levels decreased in EtOH-fed Sct^−/−^ compared to EtOH-fed WT mice. There was increased expression of Cyp27a1, Cyp8b1, Cyp7b1, Bsep, NTCP and Osta in total liver from EtOH-fed WT compared to control mice, which decreased in EtOH-fed Sct^−/−^ compared to EtOH-fed WT mice.

**Conclusions:**

Targeting Sct/SR signaling may be important for modulating ALD phenotypes.

**Supplementary Information:**

The online version contains supplementary material available at 10.1186/s13578-022-00945-w.

## Introduction

Alcohol-related liver diseases (ALD) account for nearly 50% of liver-related deaths in the US, which are attributed to excessive drinking habits [1]. The molecular mechanisms regulating the pathogenesis of ALD are complex and include oxidative stress, lipotoxicity, mitochondrial dysfunction, and possible derangement of the gut-liver axis [2]. Hepatic steatosis and fibrosis are hallmarks of ALD [3, 4], and extensive ductular reaction (DR) is also observed, but the beneficial or detrimental roles of DR in the progression of ALD remain undefined [5–8]. For example, the expansion of liver progenitor cells (associated with ALD-induced DR) correlates positively with the severity of liver disease and short-term mortality in patients with alcohol-induced hepatitis (AH) [7]. Supporting a beneficial role for DR in the modulation of liver phenotypes, a study demonstrated that neo-ductular progenitor cells regenerate hepatocytes damaged by ethanol (EtOH) [5]. Although DR is observed in several mouse models of liver damage, such as primary sclerosing cholangitis (PSC), primary biliary cholangitis (PBC), non-alcoholic fatty liver disease (NAFLD), non-alcoholic steatohepatitis (NASH), and extrahepatic cholestasis, there is not a well-defined consensus on whether DR has a beneficial or detrimental role in ALD [8–10].

The mechanisms leading to deranged bile secretion (i.e., cholestasis) and compensatory DR during ALD are undefined. Changes in bile duct permeability, compression of small biliary branches, microtubule disassembly, and impaired hepatocyte bile acid (BA) extrusion are potential alterations that might explain ALD-related cholestasis [11]. Several gastrointestinal hormones, neuropeptides, and other biological molecules modulate biliary functions [8, 12–15]. Most important among these factors is secretin (Sct), which is only expressed/secreted by cholangiocytes in the liver [12] and is considered a principal regulator of biliary secretion, senescence, and DR [16–19]. The effects of Sct are mediated by interaction with basolateral secretin receptor (SR, expressed only by cholangiocytes in the liver) [20], which leads to increased intracellular adenosine 3',5'-cyclic monophosphate (cAMP) levels, protein kinase A phosphorylation, and opening of cystic fibrosis transmembrane conductance regulator (CFTR) enhancing HCO_3_^−^ secretion through activation of anion exchanger 2 (AE2) [21–24].

There is growing information regarding the changes in the levels of Sct and the expression of the Sct/SR/miRNA-125b axis regulating biliary senescence, DR, and hepatobiliary injury [9, 16]. For example, in pathological states associated with enhanced biliary damage/DR there is enhanced expression of Sct/SR signaling and Sct-induced bicarbonate-rich choleresis [16]. In contrast, during damage/loss of bile ducts there is reduced SR signaling and Sct-stimulated bile secretion [16]. Furthermore, increased Sct signaling triggers biliary senescence/DR, and liver fibrosis by autocrine/paracrine pathways through upregulation of transforming growth factor β1 (TGFβ1) signaling [9]. In mouse models of extrahepatic obstruction by bile duct ligation (BDL) and PSC (multidrug resistance 2 knockout mice, Mdr2^−/−^), there is a marked increase in DR accompanied by enhanced biliary senescence and TGFβ1 expression that activate hepatic stellate cells (HSCs) by paracrine pathways [9, 10]. Similarly, in an early-stage PBC mouse model and human samples, there are increased levels of Sct in serum and bile and upregulation of Sct/SR signaling. In contrast, late-stage PBC models and human samples are characterized by ductopenia [25] accompanied by loss of AE2 and reduced ductal HCO_3_^−^ secretion (i.e., defective bicarbonate “umbrella”) as well as reduced Sct levels and Sct signaling [13, 15, 25–28]. Several studies have shown that inhibition of Sct-dependent signaling in BDL and Mdr2^−/−^ mice (by genetic knockout or pharmacological inhibition of SR) reduces biliary damage/DR and liver fibrosis by inhibiting TGFβ1 signaling [9, 17]. Furthermore, inhibition of biliary Sct/SR signaling (associated with reduced DR) inhibits hepatic steatosis by down-regulation of the lipid biosynthesis gene elongation of very-long-chain-fatty acids (Elovl1) through increased biliary miRNA-125b coupled with decreased angiogenesis [29]. These findings demonstrate that a proper balance in Sct levels and Sct-dependent signaling is critical to maintaining biliary homeostasis, since upregulation of Sct-dependent signaling triggers DR/biliary senescence. In contrast, Sct/SR axis reduction likely induces biliary loss/ductopenia [9, 13, 16, 17, 30]. With this background and, since alcohol-related impairment of bile secretion is undefined, we aimed to assess the role of the Sct/SR/miR-125b signaling axis in a murine model of ALD-induced cholestasis and human samples from healthy control livers and patients with alcoholic cirrhosis.

## Materials and methods

### Materials

Unless otherwise indicated, reagents were purchased from Sigma-Aldrich Chemical Co. (St. Louis, MO). Cell culture reagents and media were obtained from Invitrogen Corporation (Carlsbad, CA). Commercially available ELISA kits to measure Sct levels in mouse and human serum samples were purchased from Phoenix Pharmaceuticals (Burlingame, CA). Total RNA was extracted using the mirVana miRNA Isolation Kit from ThermoFisher Scientific (Waltham, MA). The iScript cDNA Synthesis Kit and iTaq Universal SYBR Green Supermix were purchased from Bio-Rad (Hercules, CA). All mouse and human primer information for quantitative polymerase chain reaction (*q*PCR) is listed in Additional file 3: Table S1. The list of the mouse and human antibodies used is shown in Additional file 4: Table S2.

### Animal models

Animal procedures were performed following protocols approved by the Indiana University School of Medicine Institutional Animal Care and Use Committees. C57BL/6 J wild-type (WT) mice were purchased from The Jackson Laboratory (Bar Harbor, ME). The Sct^−/−^ mouse colony (on a C57BL/6 background) [31] is established in our facility. Male WT and Sct^−/−^ mice (at 14 wk of age) were fed a Lieber-DeCarli EtOH liquid diet (5% vol/vol EtOH) for 8 wk and gavaged once per wk with 31.5% vol/vol EtOH (5 gm/kg body weight, BW) or 45% wt/vol maltose dextrin, for isocaloric equivalence, throughout the 8 wk feeding period [32]. All mice were subjected to acclimatization beginning with a 1% EtOH diet and increasing 1% each day until reaching 5% vol/vol EtOH. Six hours before sacrifice and serum, liver samples, cholangiocyte and hepatocyte collection, the mice were given a single gavage in the morning with 31.5% vol/vol EtOH or 45% wt/vol maltose dextrin as described above. All mice were housed in a temperature-controlled environment (22 ℃) with 12:12-h light–dark cycles. In all groups, body weight (BW), liver weight (LW) and LW to BW ratio (an index of liver growth) [23] were measured (Table 1). The EtOH and control groups were given supplemental nesting materials as suggested by the Indiana University School of Medicine Laboratory Animal Resource Center veterinary staff to assist in body heat retention and reduce the possibility of EtOH-induced hypothermia and/or increased mortality.

**Table 1:** Measurement of liver and body weight, liver to body weight (LW/BW) ratio, liver steatosis, inflammation and total liver bile acid (BA) levels

Treatment (mice)	Liver weight, g	Body weight, g	LW/BW ratio, %	Steatosis, %	Inflammation, %	TBA, mM
WT CD	1.30 ± 0.08 (n = 6)	29.86 ± 1.15 (n = 6)	4.33 ± 0.16 (n = 6)	0.8 ± 0.84 (n = 6)	0.60 ± 0.89 (n = 6)	18.54 ± 3.19 (n = 9)
Sct^−/−^ CD	1.77 ± 0.20 (n = 4)	38.34 ± 2.30 (n = 4)	4.57 ± 0.30 (n = 4)	1.33 ± 0.58 (n = 4)	1.00 ± 0.00 (n = 4)	16.23 ± 2.62 (n = 7)
WT EtOH	2.17 ± 0.21^a^ (n = 4)	30.68 ± 1.73 (n = 4)	7.03 ± 0.33^a^ (n = 4)	2.60 ± 0.55^a^ (n = 5)	1.80 ± 0.45^a^ (n = 5)	32.84 ± 7.02 (n = 10)
Sct^−/−^ EtOH	1.24 ± 0.13^b^ (n = 5)	29.37 ± 1.58 (n = 5)	4.19 ± 0.25^b^ (n = 5)	1.25 ± 0.50^b^ (n = 3)	1.00 ± 0.00^b^ (n = 3)	15.01 ± 3.08^b^ (n = 9)

^a^*p* < 0.05 vs WT CD

^b^*p* < 0.05 vs WT EtOH

### Human samples

Human samples from healthy control livers (n = 9) were purchased from Sekisui XenoTech (Kansas City, KS). Additionally, liver samples from healthy controls (n = 5) and patients with alcoholic cirrhosis (n = 19), as well as serum samples from healthy controls (n = 6) and patients with alcoholic cirrhosis (n = 50), and bile from healthy controls (n = 9) and patients with alcoholic cirrhosis (n = 9), were obtained from Dr. Burcin Ekser (co-author in the study) under a protocol approved by the Institutional Review Board (IRB) at Indiana University School of Medicine. The demographic characteristics of the human samples are listed in Additional file 5: Table S3 and Additional file 6: Table S4. In the Figures we use term control(s) to indicate human healthy control livers.

### Measurement of Sct/SR/CFTR/AE2 Immunoreactivity/Expression in Liver Sections, Sct Mouse and Human Serum Levels, and Bicarbonate Bile Levels in Healthy Controls and Patients with Alcoholic Cirrhosis

We evaluated the biliary immunoreactivity of: (i) Sct, SR and CFTR by immunohistochemistry in mouse liver sections (5 µm thick); and (ii) Sct, by immunohistochemistry, and SR, CFTR and AE2 by immunohistochemistry and/or immunofluorescence in liver sections (paraffin-embedded, 5 µm thick) or (frozen, 4 µm thick, co-stained with the biliary marker, CK19) [17] from healthy controls and patients with alcoholic cirrhosis. Following immunohistochemistry, sections were evaluated with an Olympus BX-51 light microscope and imaged by a DP27 Microscope Digital Camera from Olympus (Tokyo, Japan); observations were quantified with Image-Pro 10 software (Media Cybernetics, Inc. Rockville, MD) in a coded fashion. Following immunofluorescent staining, sections were mounted with coverslips using antifade gold-containing 4 V,6-diamidino-2-phenylindole (DAPI, counterstain, Molecular Probes, Eugene, OR). Images were obtained using a Leica TCS SP8 laser scanning confocal microscope (Leica Microsystems Inc., Buffalo Grove, IL).

To support the immunohistochemical data in liver sections, we measured the: (i) mRNA expression of Sct and SR in isolated mouse cholangiocytes by *q*PCR; and (ii) protein expression of SR in protein (10 mg) in total liver lysate from human healthy controls and patients with alcoholic cirrhosis by Western blotting. We used glyceraldehyde-3-phosphate dehydrogenase (GAPDH) as housekeeping for *q*PCR analysis and loading controls for Western blotting. Protein bands were detected by enhanced chemiluminescence (ECL) using a Pierce ECL Western blot substrate (Thermo Fisher Scientific) and visualized by the BioRad ChemiDoc Imaging System (BioRad Laboratories, Hercules, CA) and analyzed by Image J (normalized by GAPDH).

The ELISA kits to measure the levels of Sct in human (EK-067-03) and mouse (EK-067-04) serum were purchased from Phoenix Pharmaceuticals, Inc. (Burlingame, CA). Bicarbonate levels in bile from healthy controls and patients with alcoholic cirrhosis were analyzed using the Radiometer ABL90 FLEX plus blood gas analyzer (Radiometer America Inc.; Brea, CA). We measured bicarbonate levels in bile from healthy controls and patients with alcoholic cirrhosis since biliary bicarbonate secretion: (i) depends on and is closely associated with changes in intrahepatic biliary mass (DR) and activation of the Sct/SR signaling axis [13, 22–24]; and (ii) decreases in conditions associated with reduced DR and ductopenic states such as late-stage PBC [15, 21, 26, 30].

### Measurement of liver histology, fibrosis and steatosis

Paraffin-embedded liver sections (4 μm thick) were processed for routine histology staining by hematoxylin and eosin (H&E) and Sirius Red/Fast Green. The degree of steatosis (0 =  < 5%, 1 = 5–33%, 2 = 34–66%, and 3 =  > 66%) and lobular inflammation (0 =  < 0.5 foci per field at 20x; 1 = 0.5–1.0; 2 = 1.0–2.0 foci; 3 =  > 2.0 foci) were assessed by a semiquantitative scoring system [33]; observations were processed in a blinded fashion (see Table 1 and Additional file 1: Fig. S1).

Collagen deposition was evaluated in paraffin-embedded liver sections (4 µm thick) stained with Sirius Red/Fast Green [9]; stained slides were scanned by a digital scanner (Aperio Scanscope CS System, Aperio Digital Pathology, Leica Biosystems) and processed by QuPath (a bioimage analysis software) [34, 35]. Fibrosis extent was calculated on the entire liver section by an algorithm and expressed as the area occupied by Sirius Red-positive fibers with respect to the total areas. Immunofluorescence for desmin (a marker of HSCs) was performed in frozen liver sections (4 μm thick) co-stained with cytokeratin 19 (CK19) [18]. Immunofluorescence was visualized using the SP8 confocal microscope platform (Leica Microsystems Inc.).

### Measurement of DR, cholangiocyte and hepatocyte senescence and phenotypic switch of cholangiocytes and hepatocytes

DR was evaluated in paraffin-embedded liver sections (5 µm thick) by immunohistochemistry for CK19 [12]. Stained slides were scanned by a digital scanner (Aperio Scanscope CS System, Aperio Digital Pathology, Leica Biosystems, Milan, Italy) and processed by ImageScope. DR was calculated on the entire section by an image analysis algorithm (ImageScope) and expressed as the area occupied by CK19-positive bile ducts/total area [36].

Hepatic senescence was evaluated in frozen mouse and human liver sections (10 μm thick) by staining for senescence-associated-β-galactosidase (SA-β-gal) using the cellular senescence assay kit (MilliporeSigma, Billerica, MA); following staining sections were scanned by a digital scanner (Aperio Scanscope CS System, Aperio Digital Pathology, Leica Biosystems) and processed by ImageScope. A semi-quantitative scoring system was applied for quantification of SA-β-gal positive cholangiocytes (0 =  < 5%; 1 = 6–10%; 2 = 11–30%; 3 = 30–50%; 4 =  > 50%) [14, 18] in mouse and human samples. We confirmed cholangiocyte and hepatocyte senescence by immunofluorescence for cyclin-dependent kinase inhibitor 2A (p16), co-stained with CK19 or the hepatocyte marker, HNF4α [29] in frozen liver sections (4 μm thick). We measured the mRNA expression of the senescence gene cyclin-dependent kinase inhibitor 2C (p18) in human total liver samples by *q*PCR.

To determine if hepatocytes (damaged by EtOH feeding) acquire biliary markers, we performed immunofluorescence for CFTR and AE2 in frozen human liver sections (4 μm thick, co-stained with HNF4α) from healthy controls and patients with alcoholic cirrhosis. To determine if cholangiocytes differentiate into hepatocytes or acquire hepatocyte markers, we performed immunofluorescence for the hepatocyte bile salt export pump (BSEP) [37] in frozen mouse (WT fed CD or EtOH groups) and human liver sections (4 μm thick, co-stained with CK19) from healthy controls and patients with alcoholic cirrhosis.

### Measurement of liver inflammation and neutrophil infiltration

In paraffin-embedded mouse and/or human liver sections (4 μm thick), we evaluated hepatic inflammation by immunohistochemistry for F4/80 (marker of macrophages) [38] and cluster of differentiation 68 (CD68, a protein highly expressed by circulating macrophages) [39]; 5 different fields were analyzed for each group. Slides were scanned with a digital scanner (Aperio Scanscope CS System; Aperio Digital Pathology, Leica Biosystems) and processed with the QuPath software [35]. The number of positive macrophages was automatically quantified by an algorithm and expressed as the area fraction occupied by positive cells (%) with respect to the total parenchymal area.

The mRNA expression of the inflammatory marker chemokine ligand 1 (CXCL1) was measured in human total livers by *q*PCR. Neutrophil infiltration was measured by immunohistochemistry for myeloperoxidase (MPO) in paraffin-embedded mouse and human liver Sections (4 μm thick) and *q*PCR for lymphocyte antigen 6 complex, locus G5C (Ly6G) in human total liver samples [40, 41].

### Measurement of hepatic Cyp4a10, and Cyp4a11/22 in mouse and human liver sections

We measured the immunoreactivity of the following enzymes by which EtOH is metabolized in the liver [42]: (i) the mouse cytochrome P450, family 4, subfamily a, polypeptide 10 (Cyp4a10) in frozen mouse liver sections  (4 μm thick); and (ii) the human homologous Cyp4a11/22 [42], by immunohistochemistry (paraffin-embedded) and immunofluorescence (frozen) in human liver sections (4 μm thick). Immunofluorescent staining was performed in mouse and human frozen liver (4 μm thick) sections co-stained with CK19 or HNF4α. Immunofluorescence was visualized and quantified by the SP8 confocal microscope platform (Leica Microsystems Inc.). Since the expression of Cyp4a10 increases with EtOH feeding [42], we performed *q*PCR to determine the quantitative expression of Cyp4a10 in isolated mouse hepatocytes and cholangiocytes.

### Measurement of Sct-dependent Elovl1, total liver ba levels, and mRNA expression of genes regulating BA transport, metabolism and lipogenesis

The rationale for measuring Sct-dependent miR-125b is based on the fact that: (i) there is reduced expression of miR-125b in mouse models and human samples of early-stage PBC, PSC and NAFLD [12, 13, 17, 29]; and (ii) secretin triggers biliary damage through direct downregulation of miR-125b expression [12, 13, 17, 29]. The rationale for performing these experiments is based on our previous study showing that the Sct/miR-125b-dependent signaling axis promotes liver steatosis in mouse models of NAFLD by the up-regulation of the lipid biosynthesis gene, elongation of very-long-chain fatty acids 1 (Elovl1) [29]. We measured: (i) the expression of miR-125b in mouse total liver samples by *q*PCR [29]; and (ii) the immunoreactivity of Elovl1 by immunohistochemistry and/or immunofluorescence in mouse and human liver Sections (4 µm thick) co-stained with CK19 or HNF4α.

As an index of the degree of cholestasis, we measured the levels of hepatic total BAs (by a commercially available kit, catalog # 80,461, Crystal Chem, Elk Grove Village, IL) in total liver samples from WT and Sct^−/−^ mice fed either CD or EtOH. We have also measured the mRNA expression of genes regulating BA synthesis such as cholesterol 7a-hydroxylase (Cyp27a1) and 12a-hydroxylase (Cyp8b1), and the BA transporters, bile salt export pump (Bsep) and Na^+^-taurocholate cotransporting polypeptide (NTCP) in total mouse liver. The organic solute transporter alpha (OSTα) was evaluated by immunofluorescence in frozen mouse liver sections (4 mM thick) co-stained with CK19 or HNF4α.

### Measurement of angiogenesis

Since Sct-dependent miR-125b regulates biliary functions through changes in vascular endothelial growth factor-A (VEGF-A) [12], we measured: (i) by immunohistochemistry the immunoreactivity of VEGF-A, platelet endothelial cell adhesion molecule (CD31), Roundabout 1 (Robo1, a receptor expressed by vascular endothelial cells that promotes angiogenesis and is linked to changes in DR), Slit2 in mouse and human liver sections (4 μm thick); (ii) by immunofluorescence the immunoreactivity of von Willebrand factor (vWF) and Robo1 in mouse frozen liver sections (4 μm thick) co-stained with CK19; and (iii) the mRNA expression of vWF in human total liver samples by *q*PCR.

### Statistical analysis

All data are expressed as the mean ± SEM. Differences between groups were analyzed by unpaired Student’s t-test when two groups were analyzed and one-way ANOVA when more than two groups were analyzed, followed by an appropriate post hoc test. The level of significance was set at *P* < 0.05. For data analysis GraphPad Prism Version 9.3.1 was used.

## Results

### Measurement of Sct/SR/CFTR/AE2 immunoreactivity/expression in liver sections, Sct mouse and human serum levels, and bicarbonate bile levels in healthy controls and patients with alcoholic cirrhosis

**Fig. 1:**
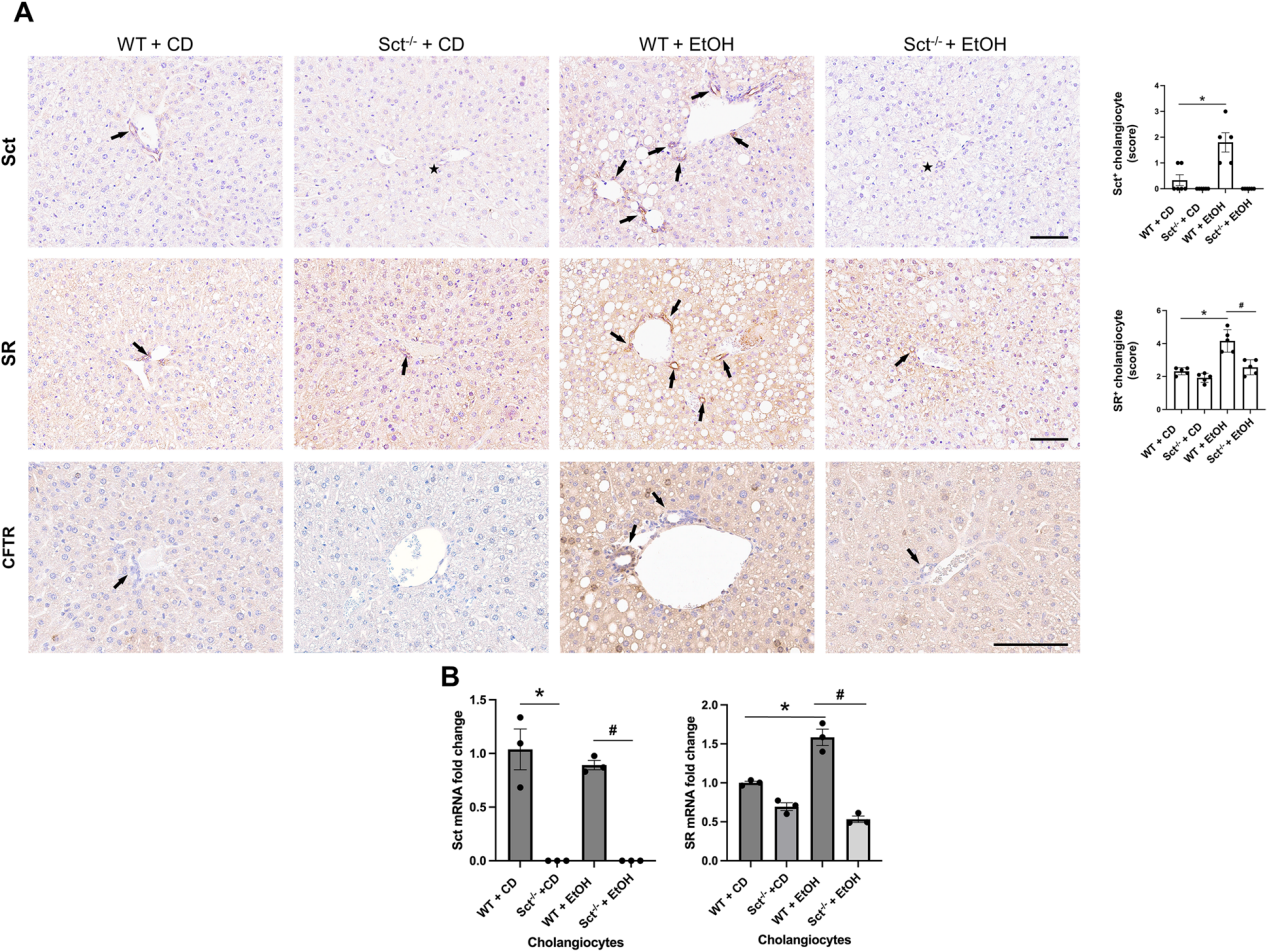
**A** Sct, SR, and CFTR immunoreactivity significantly increased in EtOH-fed WT mice compared to CD-fed WT mice. The immunoreactivity of SR and CFTR decreased in EtOH-fed Sct^−/−^ mice compared to EtOH-fed WT mice. Representative immunohistochemical images for Sct, SR, and CFTR in liver sections from CD-fed WT mice (n = 6), CD-fed Sct^−/−^ mice (n = 4), EtOH-fed WT mice (n = 5), and EtOH-fed Sct^−/−^ mice (n = 3) are shown. Orig. magn., 20X, scale bar: 100 μm. Immunohistochemical quantification of Sct and SR in mouse liver sections. Data are mean ± SEM of 5 random fields *p < 0.05 vs. CD-fed WT mice; ^#^p < 0.05 vs. EtOH-fed WT mice. Each dot represents one value in data set. Black arrows indicate bile ducts, whereas black star symbols indicate bile ducts negative for Sct. **B** Sct mRNA expression was similar between CD-fed and EtOH-fed WT mice; Sct mRNA expression was absent in CD-fed and EtOH-fed Sct^−/−^ mice. There was increased SR mRNA expression in EtOH-fed WT compared to CD-fed WT mice, which decreased in EtOH-fed Sct^−/−^ mice compared to EtOH-fed WT mice. Data are mean ± SEM of three PCR reactions from cholangiocytes from CD-fed WT mice (n = 6), CD-fed Sct^−/−^ mice (n = 4), EtOH-fed WT mice (n = 5), and EtOH-fed Sct^−/−^ mice (n = 3). *p < 0.05 vs. the values of CD-fed WT mice. ^#^p < 0.05 vs. EtOH-fed WT mice

By immunohistochemistry in mouse liver sections, we observed a significant increase in the immunoreactivity of Sct, SR, and CFTR in EtOH-fed WT mice compared to CD-fed WT mice (Fig. 1A); no immunoreactivity (black star symbol) for Sct was observed in liver sections from CD- or EtOH-fed Sct^−/−^ mice (Fig. 1A). The immunoreactivity of SR decreased in EtOH-fed Sct^−/−^ mice compared to EtOH-fed WT mice (Fig. 1A). Sct mRNA expression was similar between CD-fed and EtOH-fed WT mice; as expected Sct mRNA expression was absent in CD-fed and EtOH-fed Sct^−/−^ mice (Fig. 1B). There was increased SR mRNA expression in EtOH-fed WT compared to CD-fed WT mice, which decreased in EtOH-fed Sct^−/−^ mice compared to EtOH-fed WT mice (Fig. 1B).

**Fig. 2:**
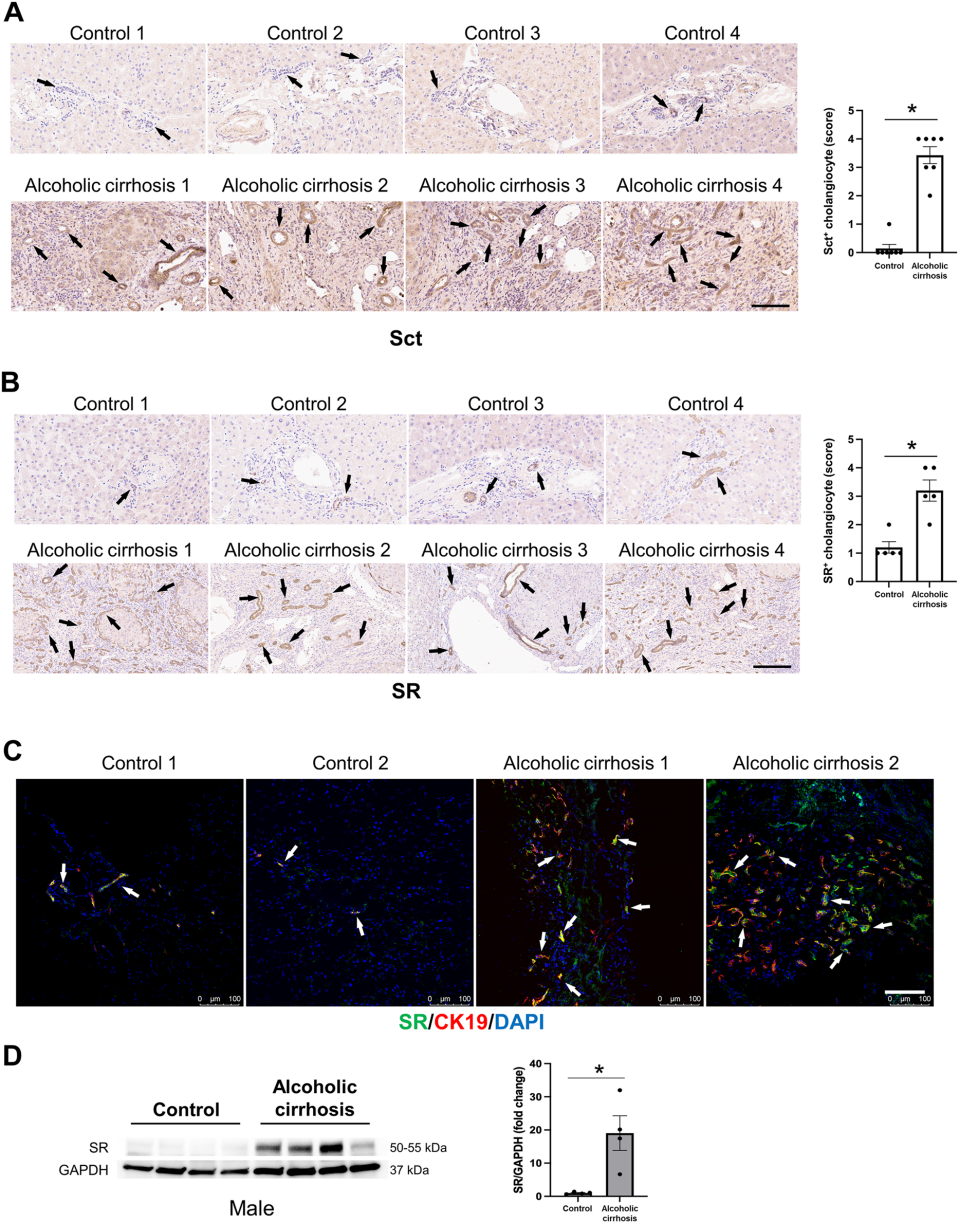
Sct and SR immunoreactivity increased in liver sections of patients with alcoholic cirrhosis compared to healthy control livers. **A** Sct and **B** SR immunoreactivity in human liver sections was examined by immunohistochemistry (4 healthy and 4 patients with alcoholic cirrhosis, from n = 8 independent samples per group. Representative images are shown at orig. magn., 20X, scale bar = 100 μm. Immunohistochemical quantification of Sct and SR in human liver sections. Data are mean ± SEM of 5 random fields. *p < 0.05 vs. respective healthy control livers. Each dot represents one value in data set. Black arrows indicate bile ducts positive for Sct or SR, whereas white arrows indicate bile ducts positive for SR. **C** Immunofluorescence in liver sections co-stained with CK19 (SR staining from 2 healthy controls and 2 patients with alcoholic cirrhosis). Representative images are shown at orig. magn. 20X, scale bar: 100 μm. **D** By immunoblots, SR protein levels increased in total liver from patients with alcoholic cirrhosis (n = 4) compared to human healthy controls (n = 4). *p < 0.05 vs. healthy control livers. Data are mean ± SEM of three separate immunoblots from total liver from patients with alcoholic cirrhosis compared to human healthy controls

**Fig. 3:**
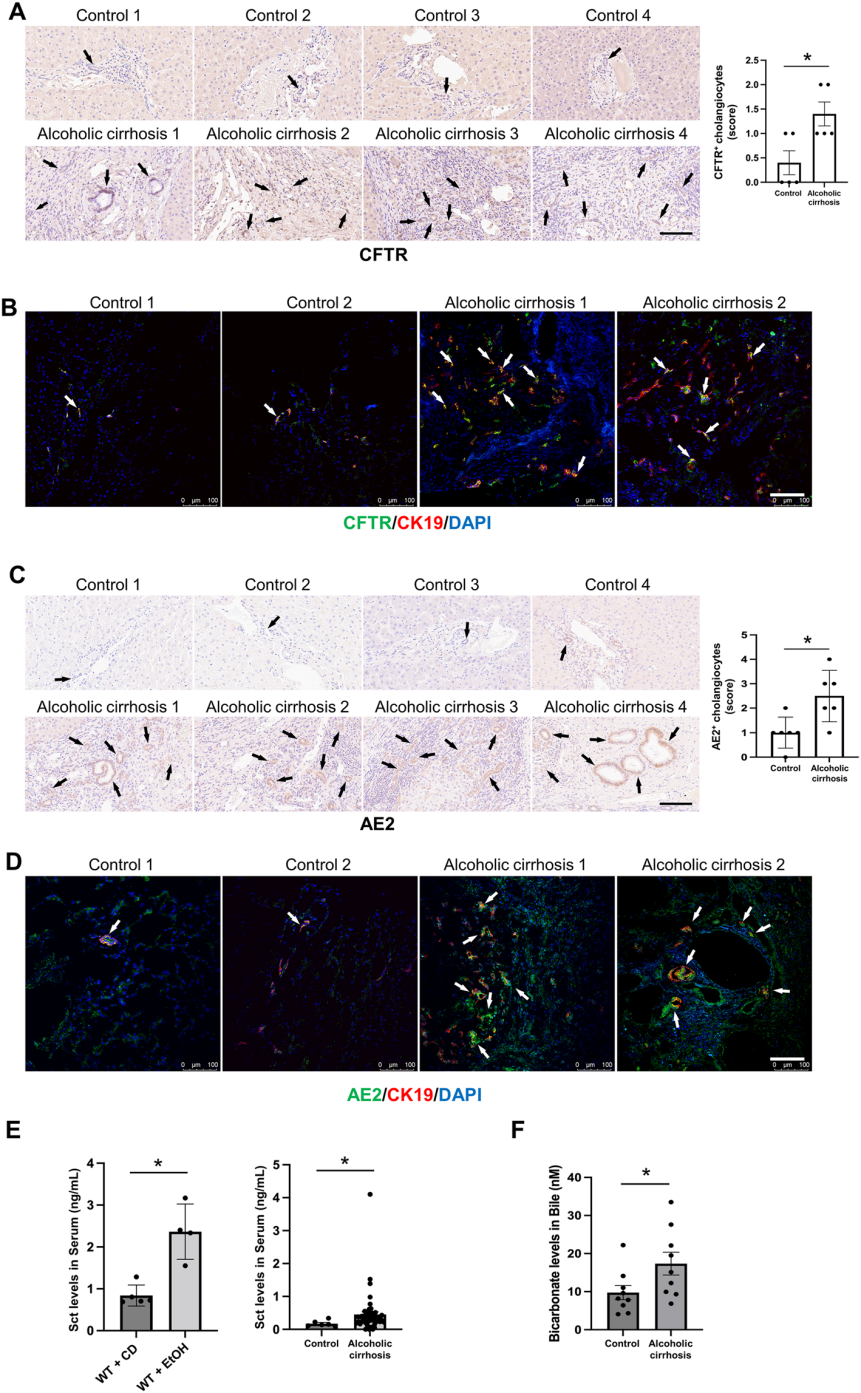
CFTR and AE2 immunoreactivity increased in liver sections of patients with alcoholic cirrhosis compared to healthy control livers. Immunoreactivity of **A** CFTR and **C** AE2 was evaluated by immunohistochemistry in liver sections from 4 healthy controls and 4 patients with alcoholic cirrhosis. Representative images are shown at orig. magn., 20X, scale bar = 100 μm. Immunohistochemical quantification of CFTR and AE2 in human liver sections. Data are mean ± SEM of 5 random fields *p < 0.05 vs. relative controls. Each dot represents one value in data set. Immunoreactivity of **B** CFTR and **D** AE2 by evaluated by immunofluorescence in liver sections co-stained with CK19 (n = 2, Orig. magn. 20X, scale bar: 100 μm) from healthy controls (n = 2) and patients with alcoholic cirrhosis (n = 2). Black and white arrows indicate bile ducts positive for CFTR and AE2. **E** Secretin levels increased in the serum from EtOH-fed WT mice and patients with alcoholic cirrhosis compared to respective control groups. Measurement of Sct serum levels from WT mice fed CD (n = 5) or EtOH (n = 4), and serum from human healthy controls (n = 6) and patients with alcoholic cirrhosis (n = 50). Data are mean ± SEM of 3 experiments. *p < 0.05 vs. the respective control mouse and human samples. **F** There was a significant increase in bicarbonate levels in bile of human patients with alcoholic cirrhosis (n = 9) compared to healthy controls (n = 9). *p < 0.05 vs. healthy control livers

By immunohistochemistry for Sct and immunohistochemistry/immunofluorescence for SR, CFTR and AE2 in human liver sections, there was increased immunoreactivity of Sct, SR, CFTR, and AE2 in patients with alcoholic cirrhosis compared to healthy control livers (Figs. 2A–C and  3A–D). By immunoblots, there was a significant increase in SR (50–55 kDa) protein expression in total liver from patients with alcoholic cirrhosis (n = 4) compared to the healthy controls (n = 4) (Fig. 2D). Sct levels were significantly higher in serum from EtOH-fed WT mice and patients with alcoholic cirrhosis compared to their respective controls (Fig. 3E). There was a significant increase in bicarbonate levels in bile of human patients with alcoholic cirrhosis (n = 9) compared to healthy controls (n = 9) (Fig. 3F).

### Depletion of Sct ameliorates liver injury and hepatic steatosis

LW/BW ratio (index of liver cell growth) [23] was higher in EtOH-fed WT mice compared to CD-fed WT mice, which was reduced in EtOH-fed Sct^−/−^ mice compared to EtOH-fed WT mice; no changes in LW/BW were observed among WT and Sct^−/−^ mice fed CD (Table 1). By H&E staining in liver sections, we observed increased steatosis and inflammation scores in EtOH-fed WT mice compared to CD-fed WT mice; these parameters were ameliorated in EtOH-fed Sct^−/−^ mice (Additional file 1: Fig. S1 and Table 1). Surprisingly, although CD-fed Sct^−/−^ mice were significantly heavier than CD-fed WT mice, these mice did not manifest significant hepatocyte steatosis. This may be due to the fact that the absence of Sct-induced bicarbonate secretion in the duodenum and small intestine of Sct^−/−^ mice favors absorption of sugar but impairs the absorption of fat [43, 44].

## Depletion of Sct reduces DR, cholangiocyte and hepatocyte senescence and phenotypic switch of cholangiocytes and hepatocytes

**Fig. 4:**
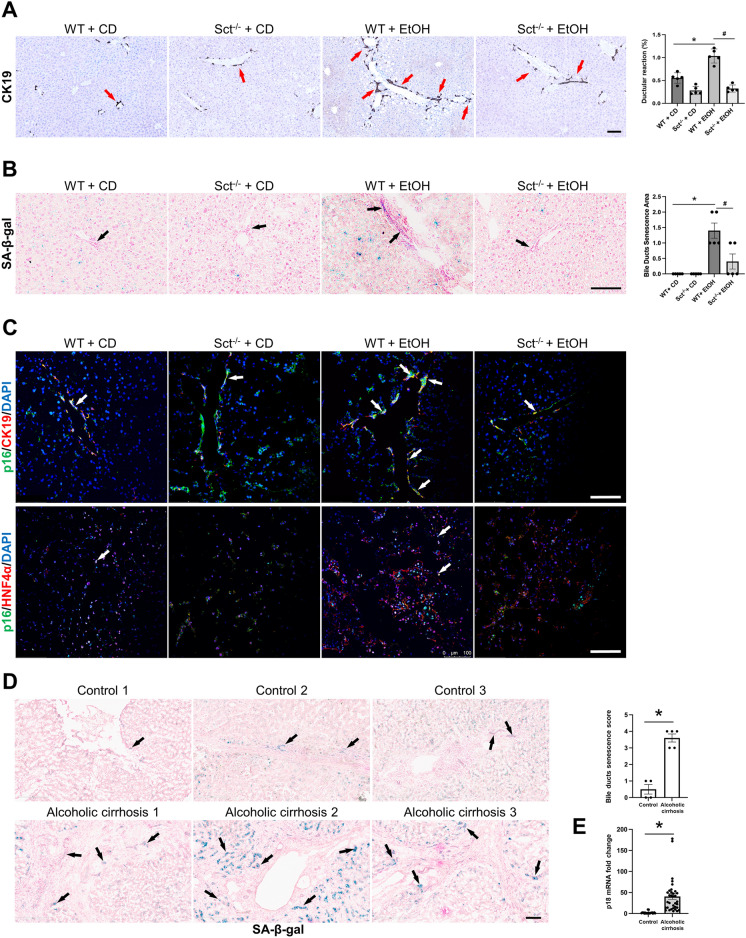
Knockout of Sct ameliorates DR and senescence in EtOH-fed mice. **A** There was a significant increase in DR in EtOH-fed WT mice compared to CD-fed WT mice, which was significantly decreased in EtOH-fed Sct^−/−^ mice compared to EtOH-fed WT mice. Data are mean ± SEM of 5 experiments from CD-fed WT mice (n = 6), WT fed-EtOH mice (n = 5), CD-fed Sct^−/−^ mice (n = 4) and EtOH-fed Sct^−/−^ mice (n = 3), Orig. magn., 20X, scale bar: 100 μm. *p < 0.05 vs. CD-fed WT mice; ^#^p < 0.05 vs. EtOH-fed WT mice. Red arrows indicate the bile ducts positive for CK19. **B**–**C** Measurement of cellular senescence in liver sections by [**B**] SA-β-gal staining (Orig. magn., 20X, scale bar: 100 μm) and **C** immunofluorescence for p16 in liver sections (co-stained with CK19 or HNF4α, respectively). Orig. magn., 20X, scale bar: 100 μm; Black arrows indicate senescent bile ducts, whereas white arrows indicate p16 positivity of cholangiocytes or hepatocytes. **D** By SA-β-Gal there was enhanced biliary senescence in liver sections from patients with alcoholic cirrhosis (n = 4) compared to human healthy controls (n = 3); orig. magn 20X, scale bar: 100 μm. Data are mean ± SEM of 5 random fields *p < 0.05 vs. healthy controls. Representative images in liver sections from patients with alcoholic cirrhosis (n = 3) and healthy controls (n = 3). Black arrows indicate senescent bile ducts. **E** By *q*PCR, there enhanced mRNA expression of p18 in total liver samples from patients with alcoholic cirrhosis (n = 16) compared to healthy controls (n = 10). Data are mean ± SEM of two experiments for each sample. *p < 0.05 vs healthy controls

There was increased DR in EtOH-fed WT mice compared to CD-fed WT mice, which was significantly decreased in EtOH-fed Sct^−/−^ mice compared to EtOH-fed WT mice; no changes in DR were observed between WT and Sct^−/−^ mice fed CD (Fig. 4A). By SA-β-gal staining, and immunofluorescence for p16 in liver sections (co-stained with CK19 or HNF4α, respectively), there was a significant increase in cellular senescence of cholangiocytes and hepatocytes in EtOH-fed WT mice compared to CD-fed WT mice; these phenotypes were significantly decreased in EtOH-fed Sct^−/−^ mice (Fig. 4B–C). We also demonstrated: (i) increased biliary senescence (by SA-β-gal staining) in human liver sections; and (ii) enhanced mRNA expression of p18 (by *q*PCR) in total liver samples from patients with alcoholic cirrhosis compared to healthy control livers (Fig. 4D–E).

**Fig. 5:**
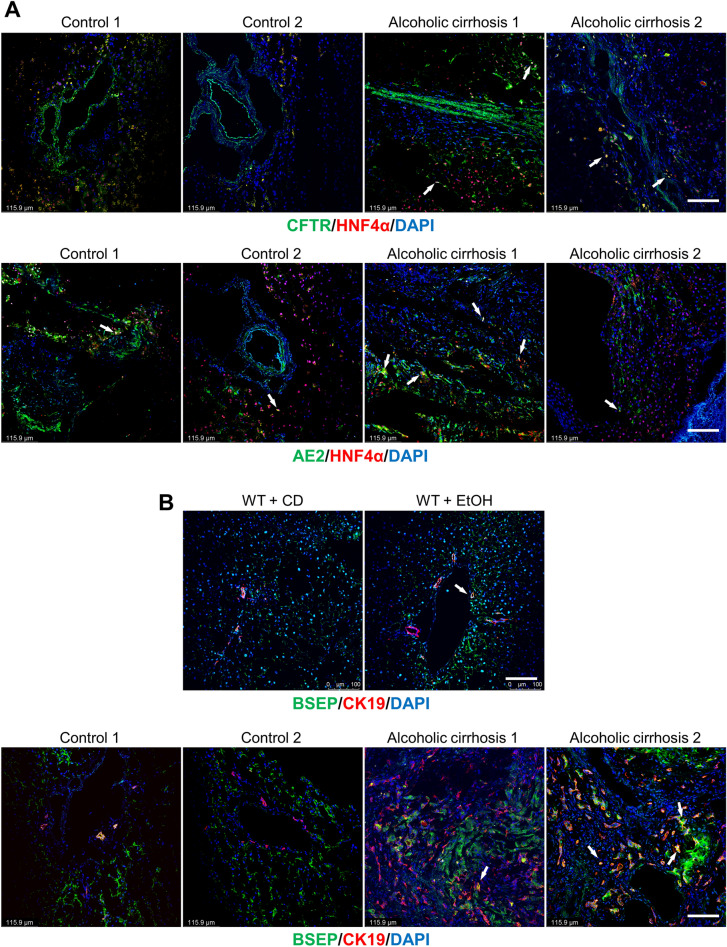
**A** Some periportal hepatocytes de novo expresses the biliary markers, CFTR and AE2 in liver sections from patients with alcoholic cirrhosis, whereas a subset of cholangiocytes de novo express BSEP in liver sections from EtOH-fed WT mice and patients with alcoholic cirrhosis; representative images from human liver sections from healthy controls (n = 2) and patients with alcoholic cirrhosis (n = 2). For immunofluorescent staining for CFTR and AE2*,* orig. magn., 20X, scale bar: 100 μm; white arrows show hepatocytes which de novo express CFTR and AE2. **B** Representative immunofluorescence images for BSEP/CK19 from CD-fed WT mice (n = 4), and EtOH-fed WT mice (n = 5), as well as human healthy controls (n = 2) and patients with alcoholic cirrhosis (n = 2), orig. magn. 20X, scale bar: 100 μm. White arrows indicate the cholangiocytes that de novo express BSEP

We also demonstrated that: (i) some periportal hepatocytes (positive for HNF4α) de novo express the biliary markers, CFTR and AE2, in liver sections from patients with alcoholic cirrhosis; and (ii) a subset of cholangiocytes de novo express BSEP in liver sections from EtOH-fed WT mice and patients with alcoholic cirrhosis (Fig. 5A–B). However, a previous study using in situ hybridization analysis demonstrated the expression of AE2 mRNA in some periportal human hepatocytes [45] suggesting that the expression of AE2 may be independent of EtOH administration. The data suggest that to compensate for the damage of a hepatocyte subpopulation (following EtOH feeding), a subset of cholangiocytes (likely small cholangiocytes that are more resistant to liver injury) [46] may acquire markers of hepatocytes and/or gradually acquire hepatocyte traits.

### Depletion of Sct reduces liver fibrosis and HSC activation

**Fig. 6:**
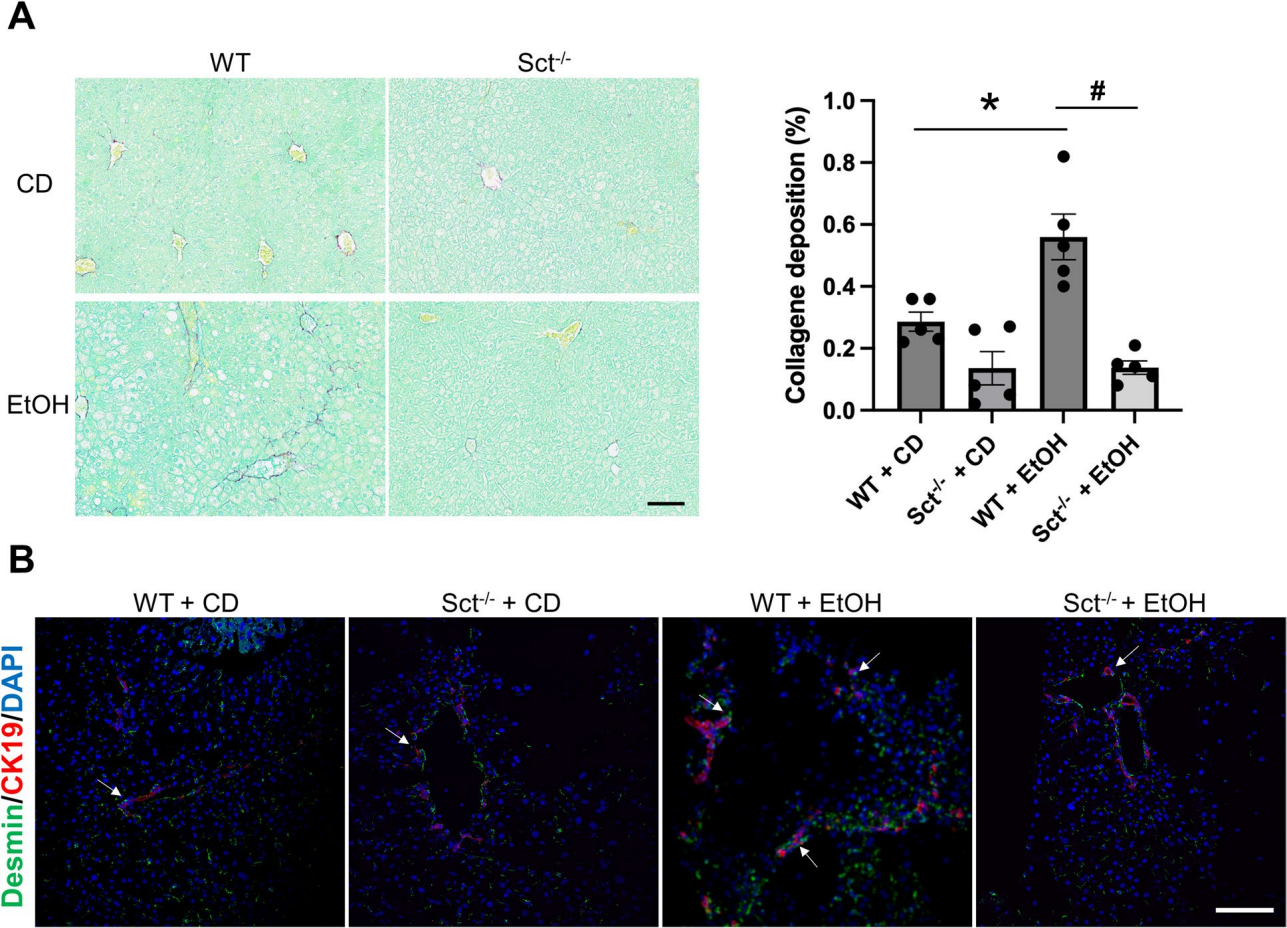
Knockout of Sct ameliorates liver fibrosis in EtOH-fed mice. **A** Measurement of collagen deposition by Sirius Red/Fast Green. Orig., magn. 20X, scale bar: 100 μm. For bar graphs, data are mean ± SEM, *p < 0.05 vs. CD-fed WT mice; ^#^p < 0.05 vs. EtOH-fed WT mice. **B** Representative immunofluorescence images for desmin in liver sections co-stained with CK19 from CD-fed WT mice (n = 6), EtOH-fed WT mice (n = 5), CD-fed Sct^−/−^ mice (n = 4), and EtOH-fed Sct^−/−^ mice (n = 4). Orig. magn. 20X, scale bar: 100 μm. White arrows indicate bile ducts positive for desmin

There was enhanced collagen deposition in liver sections from EtOH-fed WT mice compared to CD-fed WT mice, which was significantly reduced in EtOH-fed Sct^−/−^ mice compared to EtOH-fed WT mice; no changes were observed between WT and Sct^−/−^ mice fed CD (Fig. 6A). In addition, there was increased immunoreactivity of desmin in mouse liver sections (co-stained with CK19) from EtOH-fed WT mice compared to CD-fed WT mice, which decreased in Sct^−/−^ EtOH-fed mice compared to EtOH-fed WT mice (Fig. 6B). In support of our findings, a previous study has shown that the hepatic expression of the fibrotic genes, α-smooth muscle actin (α-SMA) and TGFβ1 were upregulated in EtOH-fed WT mice compared to control mice [47].

### Depletion of Sct ameliorates macrophage and neutrophil infiltration

**Fig. 7:**
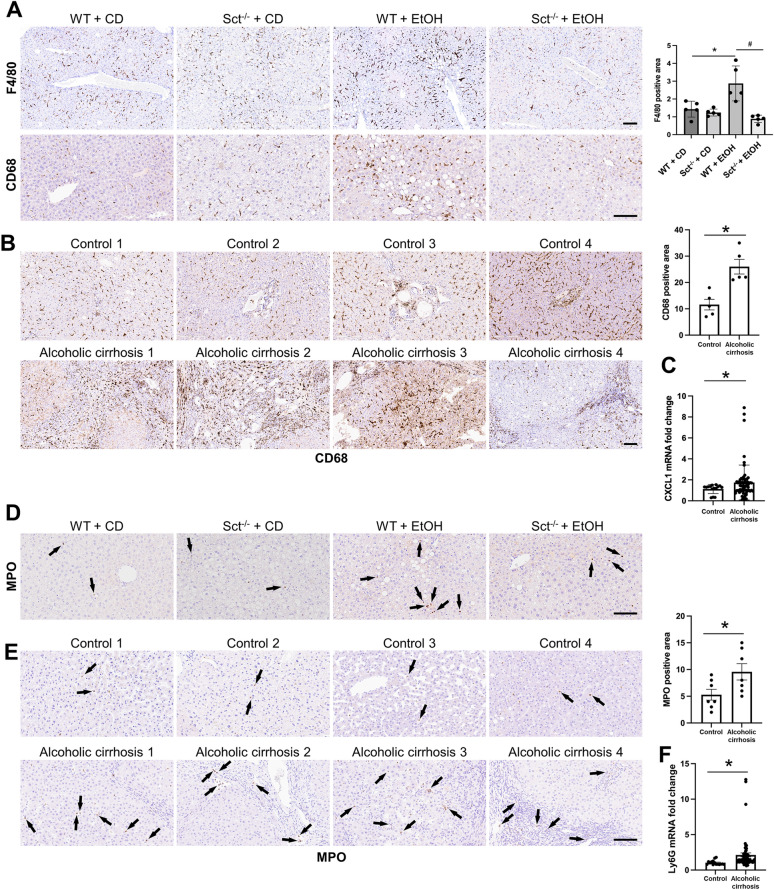
**A** Immunohistochemistry for the macrophage-specific markers, F4/80 and **B** CD68, in liver sections from the selected mouse groups (n ≥ 3/group) as well as healthy controls (n = 8), and patients with alcoholic cirrhosis (n = 8). Orig., magn. 10X; scale bar: 100 μm. For bar graphs, data are of 5 random fields mean ± SEM, *p < 0.05 vs. the respective control groups; ^#^p < 0.05 vs. EtOH-fed WT mice. **C** *q*PCR for CXCL1 in total liver samples from healthy controls (n = 6) and patients with alcoholic cirrhosis (n = 16). Data are mean ± SEM. *p < 0.05 vs. heathy control samples. **D** Immunohistochemistry for MPO in liver sections from the selected groups of mice (n ≥ 3/group), healthy controls (n = 8), and patients with alcoholic cirrhosis (n = 8). Orig. magn. 20X for mouse and 10X for human, scale bar: 100 μm. **E** Immunohistochemical quantification of MPO in human liver sections. Data are mean ± SEM of 5 random fields. *p < 0.05 vs. healthy controls. **F** *q*PCR for the neutrophil marker, Ly6G, in healthy controls (n = 6) patients with alcoholic cirrhosis (n = 16). Data are mean ± SEM of two experiments for each sample. *p < 0.05 vs healthy control samples. Black arrows indicate MPO^+^ positive cells

By immunohistochemistry in liver sections, we observed an increase in the number of (i) F4/80- and CD68-positive cells in EtOH-fed WT mice compared to CD-fed WT mice, which decreased in EtOH-fed Sct^−/−^ mice compared to EtOH-fed WT mice; and (ii) CD68 in liver samples from patients with alcoholic cirrhosis compared to the healthy control livers (Fig. 7A–B). The expression of the inflammatory marker, CXCL1, increased in total liver samples from patients with alcoholic cirrhosis compared to healthy controls (Fig. 7C). Additionally, immunohistochemistry for MPO in mouse and human liver sections showed that neutrophil infiltration was significantly increased in EtOH-fed WT mice and liver samples from patients with alcoholic cirrhosis compared to the relative control groups (Fig. 7D–E). EtOH-fed Sct^−/−^ mice displayed significantly decreased neutrophil infiltration compared to EtOH-fed WT mice (Fig. 7D). The expression of Ly6G (neutrophil infiltration marker) increased in liver samples from patients with alcoholic cirrhosis compared to healthy control livers (Fig. 7F).

### Measurement of hepatic Cyp4a10 in mouse and Cyp4a11/22 in human samples

**Fig. 8:**
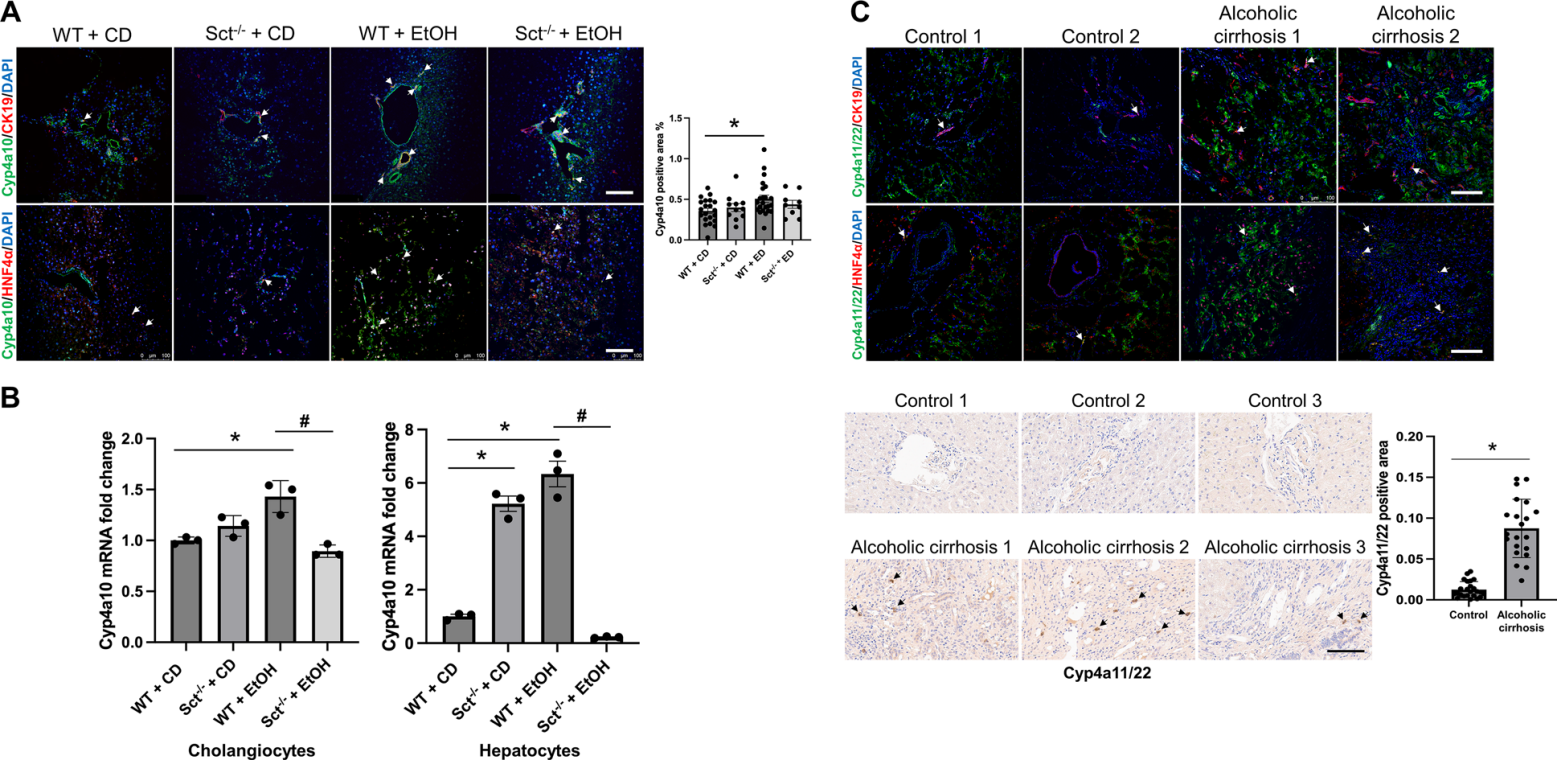
Cyp4a10 and Cyp4a11/12 are expressed by mouse and human cholangiocytes and hepatocytes, respectively. **A** Measurement of immunoreactivity for Cyp4a10 in liver sections (co-stained with CK19 or HNF4α) from selected mouse groups (n ≥ 3/group) by immunofluorescence. Orig. magn. 20X, scale bar: 100 μM. There was enhanced biliary immunoreactivity of Cyp4a10 in EtOH-fed WT mice compared to CD-fed WT mice. The Cyp4a10 positive area % refers to both cholangiocytes and hepatocytes immunoreactive for Cyp4a10. *p < 0.05 vs. CD-fed WT mice; ^#^p < 0.05 vs. EtOH-fed WT mice. **B** In EtOH-fed WT mice, there was enhanced expression of Cyp4a10 (in isolated hepatocytes and cholangiocytes) compared to CD-fed WT mice, which was reduced in EtOH-fed Sct^−/−^ mice. Data are mean ± SEM of 3 experiments from selected mice groups **(**n ≥ 3/group). *p < 0.05 vs. CD-fed WT mice; ^#^p < 0.05 vs. EtOH-fed WT mice. **C** Measurement of immunoreactivity for Cyp4a11/22 in liver sections (co-stained with CK19 or HNF4α) from human healthy controls (n = 2) and patients with alcoholic cirrhosis (n = 2) by immunofluorescence; immunoreactivity of Cyp4a11/22 was measured in paraffin-embedded liver sections from human control (n = 8) and patients with alcoholic cirrhosis (n = 8) by immunohistochemistry. Orig. magn. 20X, scale bar: 100 μm. White arrows indicate Cyp4a10- and Cyp4a11/22- positive cells. Black arrows show Cyp4a11/22-positive cells

The rationale for measuring the immunoreactivity/expression of hepatic Cyp4a10 in mouse and Cyp4a11/22 in human samples is based on the background that these enzymes are vital for the metabolism of EtOH in the liver [42]. By immunofluorescence in mouse liver sections, we demonstrated enhanced immunoreactivity of Cyp4a10 in both cholangiocytes and hepatocytes from EtOH-fed WT mice compared to CD-fed WT mice; the immunoreactivity of Cyp4a10 was not reduced in EtOH-fed Sct^−/−^ mice compared to EtOH-fed WT mice (Fig. 8A). By *q*PCR, the mRNA expression of Cyp4a10 increased in both cholangiocytes and hepatocytes isolated from EtOH-fed WT mice compared to CD-fed WT mice but decreased in EtOH-fed Sct^−/−^ mice compared to EtOH-fed WT mice (Fig. 8B). Interestingly, we observed an increase in Cyp4a10 in hepatocytes from CD-fed Sct^−/−^ mice compared to CD-fed WT mice, which may be due to a compensatory mechanism by hepatocytes due to the knock-out of Sct signaling, which may affect common transduction pathways (e.g., BA signaling) between cholangiocytes and hepatocytes. By immunofluorescence/immunohistochemistry in human liver sections, there was increased immunoreactivity of Cyp4a11/22 in patients with alcoholic cirrhosis (n = 2) compared to healthy controls (n = 2) (Fig. 8C). By immunohistochemistry there was increased immunoreactivity of Cyp4a11/22 in liver sections from patients with alcoholic cirrhosis (n = 8) compared to heathy control livers (n = 8) (Fig. 8C).

### Measurement of Sct-dependent Elovl1, total liver BA levels, mRNA expression of genes regulating BA transport, metabolism and lipogenesis

**Fig. 9:**
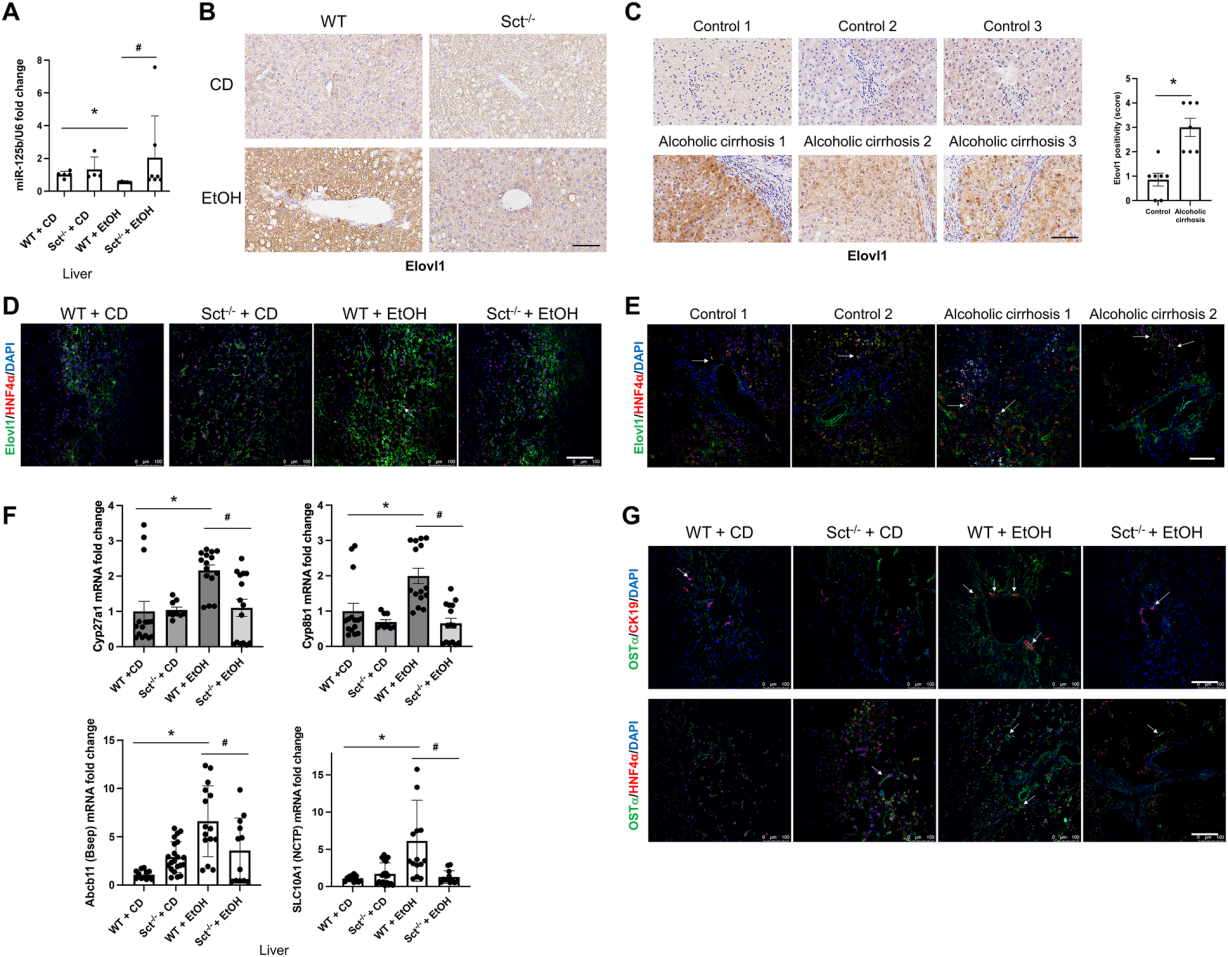
Elovl1 is expressed in hepatocytes and at low levels in cholangiocytes from mouse and human livers. **A** The expression of miR-125b in mouse liver (n ≥ 3) was evaluated by *q*PCR. For bar graphs, data are mean ± SEM of 3 replicates from CD-fed WT mice (n = 5), EtOH-fed WT mice (n = 5), CD-fed Sct^−/−^ mice (n = 5), and EtOH-fed Sct^−/−^ mice (n = 4). *p < 0.05 vs. CD-fed WT mice; ^#^p < 0.05 vs. EtOH-fed WT mice. **B**–**C** Representative images for Elovl1 in liver sections from **B** the selected mouse groups (n ≥ 3/group), **C** healthy human controls (n = 8) and patients with alcoholic cirrhosis (n = 8) by immunohistochemistry. Scale bar: 100 μM Immunohistochemical quantification of Elovl1 in human liver sections Data are mean ± SEM of 5 random fields *p < 0.05 vs. controls. **D**–**E** Immunofluorescence of Elovl1 in liver sections (co-stained with HNF4α) from **D** the selected mouse groups (n ≥ 3/group); orig. magn. 20X, scale bar: 100 μm. **E** human healthy controls (n = 2) and patients with alcoholic cirrhosis (n = 2). Orig. magn. 20X, scale bar: 100 μm. White arrows indicate Elovl1 positivity. **F** Measurement of Cyp27a1 and Cyp7b1 genes (which regulate BA synthesis), and the BA transporters genes, Bsep and NTCP, in total mouse liver. We demonstrated increased mRNA expression of Cyp27a1, Cyp8b1, Bsep and NTCP in total liver from EtOH-fed WT mice compared to CD-fed WT mice, which were decreased in EtOH-fed Sct^−/−^ mice compared to EtOH-fed WT mice. Data are mean ± SEM of three *q*PCR reactions from mouse total liver samples from CD-fed WT mice (n = 5), CD-fed Sct^−/−^ mice (n = 3), EtOH-fed WT mice (n = 5), and EtOH-fed Sct^−/−^ mice (n = 5) for Cyp27a1, Cyp8b1 mRNA expression, and CD-fed WT mice (n = 4), CD-fed Sct^−/−^ mice (n = 4; 6 experiments), EtOH-fed WT mice (n = 5), and EtOH-fed Sct^−/−^ mice (n = 4) for Bsep and NTCP mRNA expression. **G** By immunofluorescence for Ostα/CK19 and Ostα/HNF4a, there was increased immunoreactivity of Ostα in EtOH-fed WT mice compared to CD-fed WT mice, which decreased in EtOH-fed Sct^−/−^ mice compared to EtOH-fed WT mice. The selected mouse groups (n ≥ 3/group); orig. magn. 20X, scale bar: 100 μm. White arrows indicate Ostα positivity.

The rationale for measuring miR-125b in the present study is based on the fact that: (i) there is reduced expression miR-125b in mouse models and human samples of early-stage PBC, PSC and NAFLD [12, 13, 17, 29]; and (ii) Sct directly decreases the expression of miR-125b [12, 13, 17, 29]. The expression of miR-125b decreased in total liver from EtOH-fed WT mice compared to CD-fed WT mice and returned to normal values in Sct^−/−^ EtOH fed compared to EtOH-fed WT mice (Fig. 9A). By immunohistochemistry, we show that the hepatocyte immunoreactivity of Elovl1 (a lipogenesis gene targeted by miR-125b) [29] increased in liver sections from EtOH-fed WT mice compared to CD-fed WT mice but decreased in EtOH-fed Sct^−/−^ mice compared to EtOH-fed WT mice. Parallel to a previous study [29], the expression of Elovl1 was minimal in cholangiocytes (Fig. 9B). The hepatocyte immunoreactivity of Elovl1 increases in liver sections from patients with alcoholic cirrhosis (n: 8) compared to healthy control livers (n: 8) (Fig. 9C). By immunofluorescence for Elovl1/HNF4α, there was increased hepatocyte Elovl1 immunoreactivity in both mouse and human liver sections (Fig. 9D–E).

There was no significant difference in BA levels between WT mice fed CD or EtOH (18.54 ± 3.19 mM vs. 32.84 ± 7.02, respectively, p = 0.14) (Table 1); however, BA levels were reduced in EtOH-fed Sct^−/−^ mice compared to EtOH-fed WT mice (Table 1). We also demonstrated increased mRNA expression of: (i) Cyp27a1, Cyp8b1, Bsep and NTCP in total liver samples from EtOH-fed WT mice compared to CD-fed WT mice, which decreased in EtOH-fed Sct^−/−^ mice compared to EtOH-fed WT mice (Fig. 9F). By immunofluorescence for Osta/CK19 and Ostα/HNF4α, there was increased immunoreactivity of Ostα in EtOH-fed WT mice compared to CD-fed WT mice, which decreased in EtOH-fed Sct^−/−^ mice compared to EtOH-fed WT mice (Fig. 9G).

### Loss of Sct reduces angiogenesis

**Fig. 10:**
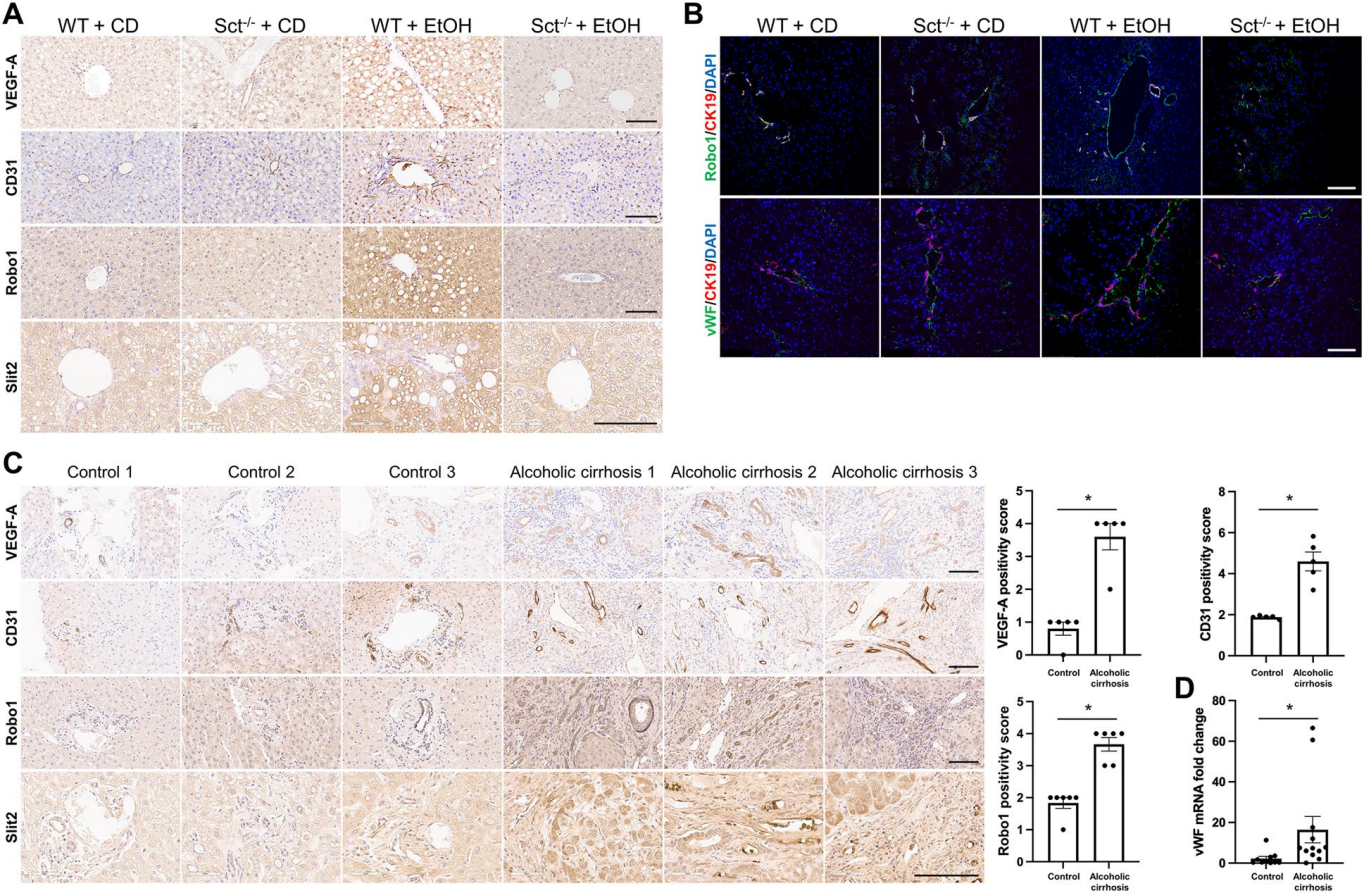
Alcohol-induced liver angiogenesis is deceased in EtOH-fed Sct^−/−^ mice. **A** Paraffin-embedded liver sections were stained with VEGF-A, CD31, Robo1, and Slit2 to evaluate liver angiogenesis in the selected mouse liver (n ≥ 3/group). Orig. magn. 20X, scale bar: 100 μM. **B** The immunoreactivity of Robo1 and vWF was evaluated in mouse liver sections co-stained with CK19 (n ≥ 3) by immunofluorescence. Orig. magn. 20X, scale bar: 100 μM. **C** The immunoreactivity of VEGF-A, CD31, Robo1, and Slit2 was examined in paraffin-embedded liver sections from human control (n = 8) and patients with alcoholic cirrhosis (n = 8) by immunohistochemistry. Orig. magn. 20X, scale bar: 100 μm Immunohistochemical quantification of VEGF-A, CD31 and Robo1 in human liver sections. Evaluation of Robo1 staining data is mean ± SEM of 5 random fields *p < 0.05 vs. Control. **D** mRNA expression of vWF was evaluated in total liver from healthy controls (n = 6) and patients with alcoholic cirrhosis (n = 16) by *q*PCR. For bar graphs, data are mean ± SEM, *p < 0.05 vs. relative controls

By immunohistochemistry, we showed increased immunoreactivity of VEGF-A and CD31 in vascular cells, Robo1 (in both bile ducts and vascular cells), and Slit2 (in bile ducts) in EtOH-fed WT mice compared to CD-fed WT mice, which was decreased in EtOH-fed Sct^−/−^ mice (Fig. 10A). Robo1 immunoreactivity was confirmed by immunofluorescence in mouse liver sections co-stained with CK19 (Fig. 10B). By immunohistochemistry the immunoreactivity of the aforementioned angiogenic factors increased in liver sections from patients with alcoholic cirrhosis (n = 8) compared to healthy control livers (n = 8) (Fig. 10C). By vWF immunofluorescence in mouse liver sections, we found enhanced angiogenesis in EtOH-fed WT mice compared to CD-fed WT mice, which was reduced in EtOH-fed Sct^−/−^ mice (Fig. 10B). By *q*PCR in total liver, we showed that the mRNA expression of angiogenesis marker vWF increased in patients with alcoholic cirrhosis compared to healthy controls (Fig. 10D).

## Discussion

In the present study, we demonstrated upregulation of Sct/SR signaling in ALD evidenced by: (i) enhanced immunoreactivity/expression of Sct/SR/CFTR in an ALD mouse model and Sct/SR/CFTR/AE2 axis in liver sections from patients with alcoholic cirrhosis; and (ii) higher levels of Sct in serum from EtOH-fed WT mice as well as patients with alcoholic cirrhosis and higher bicarbonate levels in bile from patients with alcoholic cirrhosis compared to the respective controls. We demonstrated enhanced DR, biliary senescence (that activates HSCs by a paracrine mechanism mediated by the biliary secretion of TGFβ1) [16], liver inflammation and fibrosis, infiltration of neutrophils, and liver angiogenesis in EtOH-fed WT mice compared to CD-fed WT mice, phenotypes that were reduced in EtOH-fed Sct^−/−^ mice. Parallel to the findings obtained in the mouse groups, we show enhanced DR and biliary senescence in liver samples from patients with alcoholic cirrhosis compared to healthy control livers. Concerning the expression of the enzymes metabolizing alcohol in the liver [42], we demonstrated that: (i) mouse cholangiocytes express Cyp4a10 whose immunoreactivity is increased in EtOH-fed WT mice (compared to controls) but decreased in EtOH-fed Sct^−/−^ mice. Human cholangiocytes express Cyp4a11/22, which is upregulated in the total liver from patients with alcoholic cirrhosis compared to healthy controls. EtOH-induced changes in biliary and liver phenotypes were associated with reduced biliary miR-125b expression, which facilitates the enhanced expression of hepatocyte Elovl1 expression as observed in mouse models and human samples of NAFLD [29], and subsequently ALD phenotypes.

The Sct/SR signaling axis is paramount in regulating biliary homeostasis and adaptive changes occurring during cholestasis [16]. For example, in the Mdr2^−/−^ mouse model of chronic cholestasis together with correlative experiments in human late-stage PSC liver samples, we demonstrated enhanced activity of Sct/SR signaling, which in turn activates the TGFβ1 axis triggering activation of HSCs with subsequent enhanced deposition of fibrotic matrix [9]. Recently, we have expanded these findings and evaluated the role of the biliary Sct/SR axis in liver diseases such as NAFLD and NASH. We demonstrated that Sct/SR signaling stimulates lipid accumulation via Sct-dependent downregulation of biliary miR-125b that induced lipogenesis in high fat diet (HFD)-fed mice through upregulation of hepatocyte Elovl1 [29]. Similarly, while upregulation of Sct signaling and Sct serum levels (observed in early-stage models of PBC and early-stage PBC patients) promotes DR, biliary damage and liver fibrosis [13], downregulation/loss of Sct expression/secretion (which leads to decreased bicarbonate secretion, “bicarbonate umbrella”) occurs in ductopenic diseases such as late-stage PBC [15, 21, 48]. Our studies introduce the concept that proper Sct levels maintain biliary homeostasis and liver functions. Our current findings support the idea that excessive expression levels of Sct trigger hepatobiliary damage, whereas downregulation of Sct signaling (which is detrimental in ductopenic stages) [15, 21, 48] ameliorates aberrant biliary phenotypes including senescence, liver inflammation, fibrosis, neutrophil infiltration, and angiogenesis in ALD.

Excessive consumption of alcohol is a significant cause of human liver diseases and mechanisms leading to alcohol-induced cholestasis during ALD are undefined [11]. Since cholestasis is a clinical feature of ALD [11] and Sct is a key regulator of cholestasis in liver diseases [16], we studied the role of the Sct/SR signaling axis during EtOH feeding and in human liver samples from patients with alcoholic cirrhosis. Several typical pathological features of alcohol-induced liver damage (observed in EtOH-fed WT mice) such as liver inflammation, DR, cholestasis, biliary and hepatocyte senescence, liver angiogenesis, steatosis, and fibrosis were ameliorated in Sct^−/−^ mice demonstrating an impactful relationship between Sct stimulation and the subsequent onset of pathological features after EtOH exposure. Similar to cholestatic conditions, EtOH liver damage up-regulates Sct signaling that, when chronically maintained, gives rise to DR-associated cellular senescence, which induces inflammation and fibrosis. While the increase in Sct serum levels (observed in patients with alcoholic cirrhosis) supports the concept that Sct triggers hepatobiliary damage and liver fibrosis, the increase in HCO_3_^−^ levels in bile samples (although not significant) from patients with alcoholic cirrhosis may be due to a compensatory secretory repair mechanism by adjacent non-senescent cholangiocytes which may secrete HCO_3_^−^ by Ca^2+^-dependent Cl^−^ channels (e.g., transmembrane member 16A (TMEM16A) [49] independent of cAMP signaling, however, further studies are necessary to support this concept.

After we established that downregulation of the Sct/SR signaling axis reduces liver fibrosis and steatosis with decreased DR/biliary senescence likely by paracrine signaling, we evaluated the mechanisms by which cholangiocytes metabolize EtOH inducing changes in biliary and liver phenotypes. In addition to the metabolism of EtOH by hepatocytes (mediated by Cyp4a10 [mouse] and Cyp4a11/22 [human]) [42], our data also demonstrate that cholangiocytes may metabolize EtOH through the enzymes, Cyp4a10 and Cyp4a11/22 (whose expression is increased in EtOH-fed WT mice and liver samples from patients with alcoholic cirrhosis). These biliary/liver phenotypes were ameliorated in EtOH-fed Sct^−/−^ mice, associated with a concomitant decreased expression of Cyp4a10 and Cyp4a11A22 in cholangiocytes and Cyp4a11/22 in hepatocytes [42]. Supporting our findings, several studies have emphasized the expression of metabolizing enzymes in bile ducts and the role of cholangiocytes in the clearance of liver toxins [46]. A single dose of carbon tetrachloride (CCl_4_) induces apoptosis of large cholangiocytes since they express the enzyme, cytochrome P-4502E1, which converts CCl_4_ to free-radical species such as trichloromethyl free radicals [50]. During CCl_4_-induced damage to large cholangiocytes, small cholangiocytes (which do not express cytochrome P-4502E1 and are resistant to CCl_4_-induced damage) de novo express markers of large cholangiocytes (i.e., SR) and proliferate to maintain homeostatic biliary mass [50]. Furthermore, hyperplastic cholangiocytes lining interlobular ducts of cholestatic BDL rats express Phase II (UDP-glucuronosyltransferase and glutathione S-transferase) but not Phase I enzymes. The expression of Phase II enzymes in interlobular bile ducts may alter the resistance of these ducts to the cytotoxic effects of hepatotoxins [46].

Our data suggests that changes in the expression of the Sct-dependent signaling may be necessary for the modulation of liver injury. In agreement with this concept, several studies demonstrated that the effects of the Sct/SR signaling axis on biliary damage and liver fibrosis are mediated by paracrine modulation of TGFβ1 biliary secretion that leads to activation/deactivation of HSCs and changes in liver fibrosis through modulation of liver angiogenesis [9, 16, 17]. Specifically, Sct stimulates DR and liver fibrosis (by a paracrine loop) in cholestatic models by enhanced biliary secretion of TGFβ1. In contrast, downregulation of Sct/SR signaling reduces DR/biliary senescence, liver angiogenesis and liver fibrosis by downregulation of TGFβ1 [9, 16, 17]. Consistent with our previous studies, we show that EtOH feeding triggers angiogenesis, increasing the expression of angiogenic factors (that activates liver fibrosis) in WT mice, which was decreased in EtOH-fed Sct^−/−^ mice.

We next evaluated the mechanisms by which changes in biliary Sct signaling (following EtOH feeding) may contribute to changes in hepatic steatosis through paracrine mechanisms mediated by Sct. Similar to our previous studies in a mouse model of NAFLD [29], we demonstrated that chronic EtOH feeding increases Sct levels and Sct/SR signaling that decreases the biliary expression of miR-125b, which in turn increases the expression of the lipogenesis target gene, Elovl1 (expressed mostly in hepatocytes and at low levels by cholangiocytes) triggering hepatocyte lipid accumulation and hepatic steatosis. Supporting this concept, several studies demonstrated the critical role of ELOVL family in the modulation of liver injury in mouse models of NAFLD and ALD. For example, in Pten null mouse livers, there is increased expression of Elovl1 and Elovl6, which are associated with enhanced lipogenesis [51]. The hepatocyte Elovl6 has been shown to regulate ceramide acyl-chain length and hepatic insulin sensitivity in mice linked to Pnpla3-mediated NAFLD [52]. Further, studies have shown that Elovl6 promotes NAFLD, whereas absence of Elovl6 ameliorates steatohepatitis in a lithogenic diet-fed LDL receptor-deficient mouse model [53, 54]. Moreover, Elovl6 expression increases in murine models of chronic alcohol administration [55, 56]. Additionally, Elovl5 regulates hepatic triglyceride catabolism in obese C57BL/6 J mice [57]. Future studies are needed to better demonstrate this pathway in our mouse ALD models.

Regarding angiogenesis, a number of studies suggest that cross-talk signaling between the biliary epithelium and the peribiliary vascular plexus (that nourishes the biliary epithelium) [58] coordinately modulate biliary proliferation/damage and liver fibrosis during cholestatic liver injury [13, 29, 58–60]. Our findings support the concept that liver angiogenesis (mediated by Sct/SR signaling) is an essential regulator of DR, liver fibrosis, and steatosis in cholangiopathies. Indeed, Sct effects on biliary and liver phenotypes have been shown to be mediated by changes in the expression of angiogenic factors regulated by the Sct/SR signaling axis [9, 12, 13, 16, 29]. This concept is supported by several studies showing that angiogenic factors, such as VEGF-A stimulate biliary proliferation, liver fibrosis and lipogenesis associated with increased hepatic steatosis in cholestatic models [59, 61, 62].

A limitation of our study is represented by the fact that we do not evaluate the downstream Sct-dependent signaling pathways in cholangiocytes that may partly explain the paracrine effect on hepatocyte EtOH metabolism.

The rationale for measuring the levels of total BAs and the mRNA expression of genes regulating BA synthesis and transport in mouse and human liver samples is based on the findings that there is synergistic interaction between Sct and BA signaling. For example, we have shown [15] that treatment of a late stage mouse model of PBC (dominant-negative TGFb receptor II, dnTGFbRII at 32 wk of age) with Sct for 8 wk reduced hepatic total BA content and induced in female dnTGFbRII mice a significant reduction in taurine-conjugated b-muricholic acid, an antagonist of farnesoid X receptor, which after BA activation regulates bile acid synthesis, metabolism, and transport [63]. Furthermore, treatment of male dnTGFbRII mice (32 wk age) with Sct for 8 weeks reduced the levels of taurine-conjugated cholic acid an increased taurine-conjugated chenodeoxycholic acid content; these data suggest that Sct signaling play an important role in the regulation of cholehepatic shunting and the associated total BA clearance [15]. In addition, in mice fed HFD for 16 wk, knockout of the Sct/SR signaling axis induced an increase in total bile acid fecal excretion, altered total liver BA composition and induced an increase in serum BA levels compared to WT mice fed HFD [29]. Supporting the role of Sct in the modulation of BA signaling, our current data have shown an increase in the mRNA expression genes regulating BA synthesis (Cyp27a1, Cyp8b1 and Cyp7b11), and BA transporters (Bsep, NTCP and OSTa) in liver samples from EtOH-fed WT mice compared to CD-fed WT mice, which decreased in EtOH-fed Sct^−/−^ mice compared to EtOH-fed WT mice. Supporting a possible feed-back mechanism between Sct and BAs, a study has shown that Sct has been shown to stimulate cholehepatic shunting of conjugated BAs through increased expression of the apical sodium-BA transporter (ASBT) [64]. Furthermore, the BAs ursodeoxycholate and tauroursodeoxycholate inhibit biliary proliferation, ductal secretion, cAMP levels, and ASBT expression through phosphorylation of the Ca^2+^-dependent protein kinase alpha [65]. Moreover, the BAs, cholic acid and chenodeoxycholic acid, have been shown to inhibit duodenal secretin expression through increased expression of orphan nuclear receptor small heterodimer partner [66]. A limitation of our studies is the fact that we have used total Sct^−/−^ mouse ALD models that do not exclude the possibility that changes in hepatic steatosis may be due to impaired intestinal lipid absorption; however, we have developed a cholangiocyte-specific SR^−/−^ mouse model that we will use in future experiments to pinpoint the role of biliary Sct/SR signaling on liver phenotypes [43].

Our study introduces the concept that, in addition to the direct effects of EtOH on hepatocyte functions [42], alcohol feeding induces changes in Sct-dependent biliary phenotypes which contributes to liver fibrosis and lipogenesis and subsequently hepatocyte steatosis which is in keeping with our previous study in animal models of NAFLD (29). Modulating the Sct/SR axis may be important to modulate DR/biliary senescence and liver fibrosis and regulate hepatocyte lipogenesis/steatosis and liver angiogenesis by paracrine pathways (Additional file 2: Fig. S2). In this perspective, down-regulation of the Sct/SR pathway might also benefit chronic liver diseases.

## Supplementary Information

**Additional file 1: Figure S1.** Knockout of Sct ameliorates liver damage compared to EtOH-fed WT mice. Representative images and evaluation of liver histology by H&E staining in paraffin-embedded liver sections from CD-fed WT mice (n=6), CD-fed Sct^-/-^ mice (n=4), EtOH-fed WT mice (n=5), and EtOH-fed Sct^-/-^ mice (n=3). There was increased liver damage in EtOH-fed WT mice compared to CD-fed WT mice, phenotypes that were ameliorated in EtOH-fed Sct^-/-^ mice compared to EtOH-fed WT mice. Orig. magn., 20X, scale bar = 100 μm.

**Additional file 2: Figure S2.** Working model: Alcohol consumption increases Sct/SR signaling axis in cholangiocytes, which through downregulation of miR-125 mechanism stimulates VEGF-A and trigger DR/biliary senescence, liver inflammation and fibrosis in the liver. The connection between Sect/SR signaling axis and Cyp4a10 enzyme regulates not only liver fibrosis but also hepatic steatosis through paracrine mechanisms regulated by the miR-125b/Elovl1 signaling axis inducing changes in lipogenesis. Knock-out of the Sct/SR axis ameliorates these liver phenotypes. Created with Biorender.com.

**Additional file 3: Table S1.** List of mouse and human primers for PCR.

**Additional file 4: Table S2.** List of antibodies.

**Additional file 5: Table S3.** Characteristics of healthy controls and ALD patients for liver, bile and cholangiocytes.

**Additional file 6: Table S4.** Characteristics of healthy controls and ALD patients for serum.

## Data Availability

Not applicable.

## Abbreviations

AE2: Anion exchanger 2
ALD: Alcoholic-related liver diseases
α-SMA: α-Smooth muscle actin
BA: Bile acid
BDL: Bile duct ligation
BSEP: Hepatocyte bile salt export pump
cAMP: Adenosine 3',5'-cyclic monophosphate
CD: Control diet
CD31: Platelet endothelial cell adhesion molecule
CD68: Cluster of differentiation 68
CFTR: Cystic fibrosis transmembrane conductance regulator
CK19: Cytokeratin 19
Cyp4a: Cytochrome P450 4A
CYP7A1: Cholesterol 7a-hydroxylase
Cyp8b1: 12A-hydroxylase
Cyp27a1: Sterol 27-hydroxylase
CYP7B1: Oxysterol 7a-hydroxylase
CXCL1: Chemokine ligand 1
DAPI: 4 V,6-diamidino-2-phenylindole
DR: Ductular reaction
Elovl1: Elongation of very-long-chain fatty acids 1
EtOH: Ethanol
H&E: Hematoxylin and eosin
GAPDH: Glyceraldehyde-3-phosphate dehydrogenase
HNF4 α: Hepatocyte nuclear factor α
HSCs: Hepatic stellate cells
HIBEpiC: Human normal intrahepatic biliary epithelial cell
IL: Interleukin
Ly6G: Lymphocyte antigen 6 complex, locus G
Mdr2^−^^/^^−^: Multidrug resistance protein 2
miRNA: Micro-RNA
MPO: Myeloperoxidase
MRP3: Multidrug resistance-associated protein 3
NAFLD: Nonalcoholic fatty liver disease
NASH: Non-alcoholic steatohepatitis
NTCP: Na^+^-taurocholate cotransporting polypeptide
OSTα: Organic solute transporter a
PBC: Primary biliary cholangitis
PCNA: Proliferating cellular nuclear antigen
p16: Cyclin-dependent kinase inhibitor 2A
p18: Cyclin-dependent kinase inhibitor 2C
PSC: Primary sclerosing cholangitis
*q*PCR: Quantitative polymerase chain reaction
SA-β-gal: Senescence-associated-β-galactosidase
Sct: Secretin
SR: Secretin receptor
TGFβ1: Transforming growth factor β1
VEGF-A: Vascular endothelial growth factor-A
vWF: Von Willebrand factor
WT: Wild-type
