# Supplementary figures and images for: Knockout of secretin ameliorates biliary and liver phenotypes during alcohol-induced hepatotoxicity
Source: Cell Biosci. 2023 Jan 9;13:5. doi: 10.1186/s13578-022-00945-w (PMC9830859; doi:10.1186/s13578-022-00945-w)

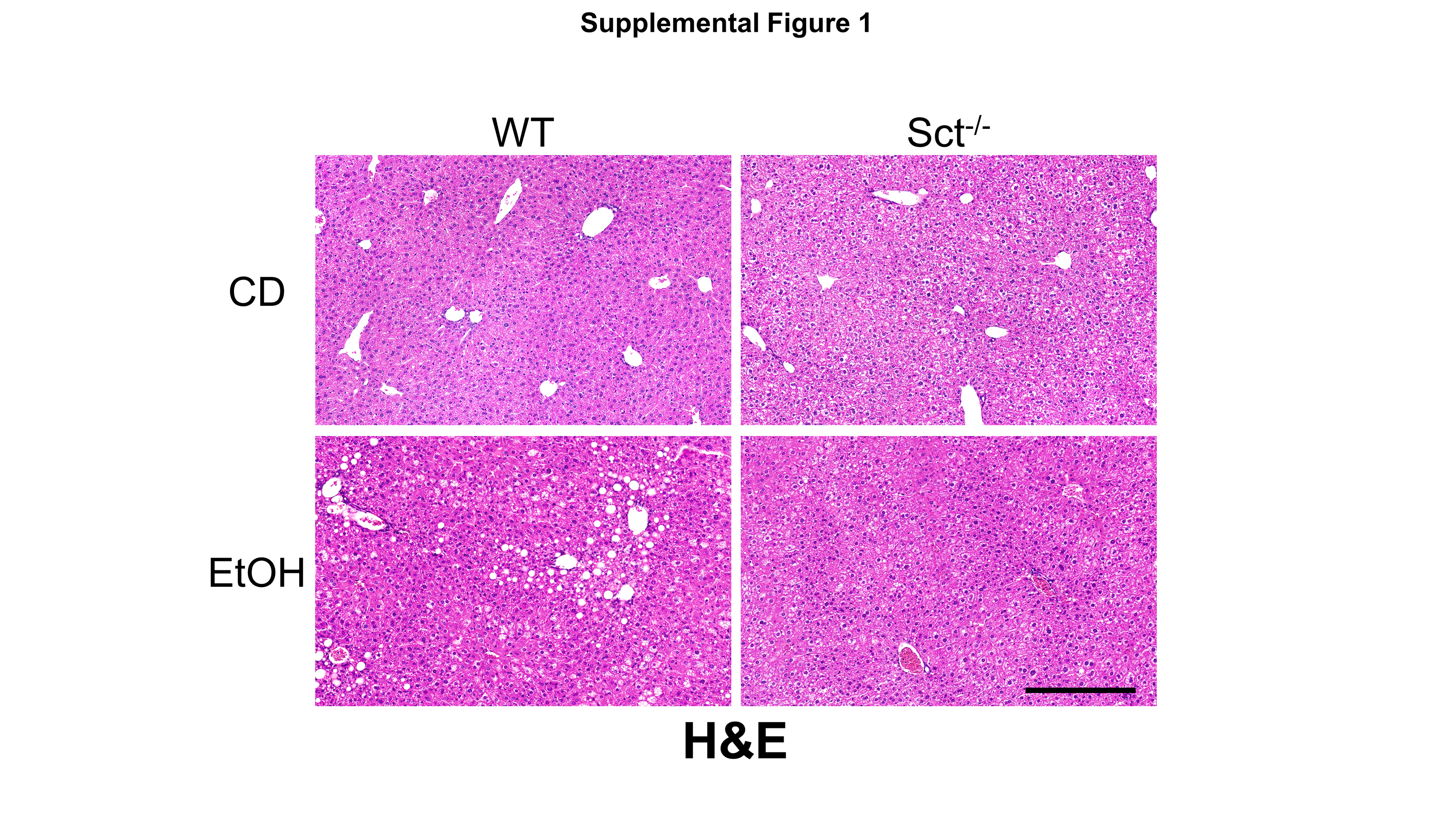

Supplement: Supplementary file 1 — Additional file 1: Figure S1. Knockout of Sct ameliorates liver damage compared to EtOH-fed WT mice. Representative images and evaluation of liver histology by H&E staining in paraffin-embedded liver sections from CD-fed WT mice (n=6), CD-fed Sct-/- mice (n=4), EtOH-fed WT mice (n=5), and EtOH-fed Sct-/- mice (n=3). There was increased liver damage in EtOH-fed WT mice compared to CD-fed WT mice, phenotypes that were ameliorated in EtOH-fed Sct-/- mice compared to EtOH-fed WT mice. Orig. magn., 20X, scale bar = 100 μm. [file 13578_2022_945_MOESM1_ESM.tif]

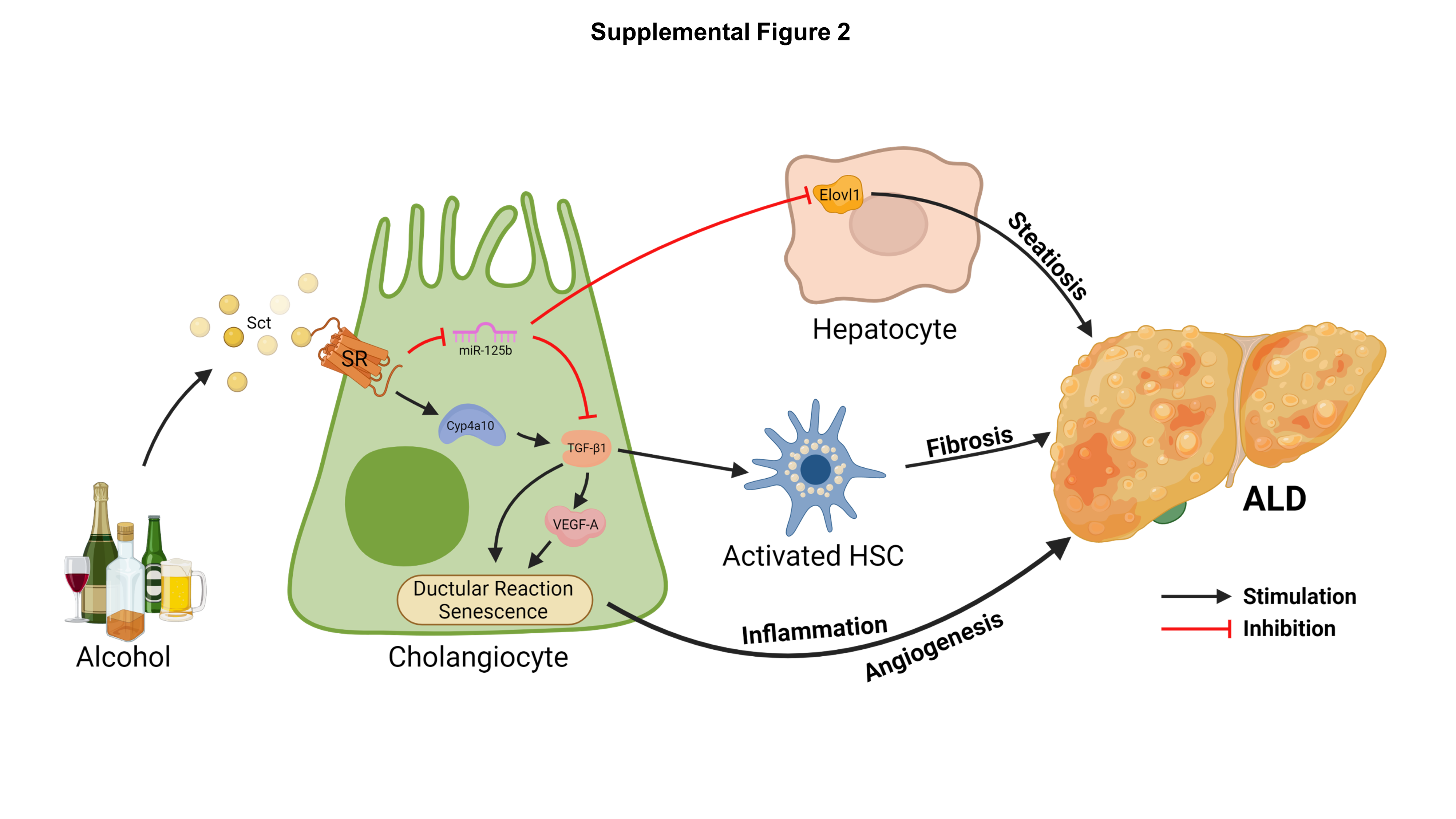

Supplement: Supplementary file 2 — Additional file 2: Figure S2. Working model: Alcohol consumption increases Sct/SR signaling axis in cholangiocytes, which through downregulation of miR-125 mechanism stimulates VEGF-A and trigger DR/biliary senescence, liver inflammation and fibrosis in the liver. The connection between Sect/SR signaling axis and Cyp4a10 enzyme regulates not only liver fibrosis but also hepatic steatosis through paracrine mechanisms regulated by the miR-125b/Elovl1 signaling axis inducing changes in lipogenesis. Knock-out of the Sct/SR axis ameliorates these liver phenotypes. Created with Biorender.com. [file 13578_2022_945_MOESM2_ESM.tif]
