# Supplementary material for: Knockout of secretin ameliorates biliary and liver phenotypes during alcohol-induced hepatotoxicity
Source: Cell Biosci. 2023 Jan 9;13:5. doi: 10.1186/s13578-022-00945-w (PMC9830859; doi:10.1186/s13578-022-00945-w)
Supplement: Supplementary file 3 — Additional file 3: Table S1. List of mouse and human primers for PCR. [file 13578_2022_945_MOESM3_ESM.docx]

**Additional file Table S1 List of mouse and human primers for PCR**

| **Gene** | **Species** | **Detected transcript** | **Source** | **GeneGlobe ID** |
| --- | --- | --- | --- | --- |
| Bsep | Mouse | NM_021022 | QIAGEN | PPM03929E-200 |
| Cyp27a1 | Mouse | NM_024264 | QIAGEN | PPM30124A-200 |
| Cyp8b1 | Mouse | NM_010012 | QIAGEN | PPM03916A-200 |
| GAPDH | Mouse | NM_008084 | QIAGEN | PPM02946E-200 |
| Ntcp | Mouse | NM_011387 | QIAGEN | PPM30690A-200 |
| p18 | Mouse | NM_007671 | QIAGEN | PPM02893C-200 |
| CXCL1 | Human | NM_001511 | QIAGEN | PPH00696C-200 |
| GAPDH | Human | NM_002046 | QIAGEN | PPH00150F-200 |
| LY6G6C | Human | NM_025261 | QIAGEN | PPH01106A-200 |
| p18 | Human | NM_078626 | QIAGEN | PPH00208B-200 |
| vWF | Human | NM_000552 | QIAGEN | PPH02567F-200 |
