# Supplementary material for: Knockout of secretin ameliorates biliary and liver phenotypes during alcohol-induced hepatotoxicity
Source: Cell Biosci. 2023 Jan 9;13:5. doi: 10.1186/s13578-022-00945-w (PMC9830859; doi:10.1186/s13578-022-00945-w)
Supplement: Supplementary file 4 — Additional file 4: Table S2. List of antibodies. [file 13578_2022_945_MOESM4_ESM.docx]

| **Antibodies** | **Application** | **Dilution** | **Description** | **Source** | **Catalog no.** |
| --- | --- | --- | --- | --- | --- |
| AE2 | IHC | 1:50 | Mouse monoclonal | Santa Cruz Biotechnology (Dallas, TX) | sc-376632 |
| AE2 | IF | 1:200 | Rabbit polyclonal | Abcam (Cambridge, MA) | ab42687 |
| BSEP | IF | 1:500 | Rabbit polyclonal | Invitrogen (Waltham, MA) | PA5-78690 |
| CD31 | IHC for mouse | 1:100 | Goat polyclonal | R&D Systems (Minneapolis, MN) | AF3628 |
| CD31 | IHC for human | 1:50 | Rabbit polyclonal | Abcam | ab28364 |
| CD68 | IHC for mouse | 1:150 | Rabbit monoclonal | Cell Signaling Technology (Danvers, MA) | 97778S |
| CD68 | IHC for human | 1:400 | Rabbit monoclonal | Cell Signaling Technology | 76437S |
| CFTR | IHC for mouse | 1:50 | Goat polyclonal | Santa Cruz Biotechnology | sc-8909 |
| CFTR | IHC for human | 1:200 | Mouse monoclonal | Abcam | ab2784 |
| CFTR | IF | 1:100 | Mouse monoclonal | Abcam | ab2784 |
| CK19 | IHC | 1:400 | Rabbit monoclonal | Abcam | ab52625 |
| CK19 | IF | 1:100 | Rat monoclonal | Developmental Studies Hybridoma Bank (Iowa City, IA) | TROMA-III |
| Cyp4a10 | IF | 1:100 | Rabbit polyclonal | Invitrogen | PA3-033 |
| Cyp4a11/22 | IHC for human | 1:100 | Rabbit polyclonal | Bioss Antibodies (Woburn, MA) | bs-5054R |
| Cyp4a11/22 | IF | 1:250 | Rabbit polyclonal | Invitrogen | PA5-102018 |
| Desmin | IF | 1:20 | Goat polyclonal | R&D Systems | AF3844 |
| Elovl1 | IHC for mouse | 1:100 | Rabbit polyclonal | Abcam | ab230634 |
| Elovl1 | IHC for human | 1:50 | Rabbit polyclonal | Novus Biologicals (Centennial, CO) | NBP2-37956 |
| Elovl1 | IF for mouse | 1:500 | Rabbit polyclonal | Abcam | ab204489 |
| Elovl1 | IF for human | 1:500 | Rabbit polyclonal | Novus Biologicals (Centennial, CO) | NBP2-37956 |
| F4/80 | IHC | 1:250 | Rabbit monoclonal | Cell Signaling Technology | 70076S |
| HNF4α | IF | 1:50 | Goat polyclonal | LifeSpan BioSciences (Seattle, WA) | LS-C758303 |
| MPO | IHC for mouse | 1:800 | Rabbit polyclonal | Abcam | ab139748 |
| MPO | IHC for human | 1:1000 | Rabbit monoclonal | Cell Signaling Technology | 14569S |
| OSTα | IF | 1:50 | Rabbit polyclonal | Biorbyt (St Louis, MO) | orb450873 |
| p16 | IF | 1:50 | Rabbit polyclonal | Abcam | ab189034 |
| Robo1 | IHC/IF | 1:250 | Rabbit polyclonal | Abcam | ab7279 |
| Sct | IHC | 1:200 | Rabbit polyclonal | Bioss Antibodies | bs-0088R |
| SLIT2 | IHC | 1:300 | Rabbit polyclonal | Invitrogen | PA5-31133 |
| SR | IHC for mouse; WB | 1:200 | Rabbit polyclonal | Bioss Antibodies | bs-0089R |
| SR | IHC for human | 1:200 | Rabbit polyclonal | Abcam | ab85565 |
| SR | IF | 1:50 | Rabbit polyclonal | Bioss Antibodies | bs-0089R |
| VEGF-A | IHC for mouse | 1:100 | Rabbit polyclonal | Abcam | ab39250 |
| VEGF-A | IHC for human | 1:100 | Rabbit monoclonal | Abcam | ab52917 |
| vWF | IF | 1:100 | Rabbit polyclonal | Abcam | ab9378 |

**Additional file Table S2 List of antibodies**

IHC, immunohistochemistry; IF, immunofluorescence; WB, Western blot.
