# Supplementary material for: Knockout of secretin ameliorates biliary and liver phenotypes during alcohol-induced hepatotoxicity
Source: Cell Biosci. 2023 Jan 9;13:5. doi: 10.1186/s13578-022-00945-w (PMC9830859; doi:10.1186/s13578-022-00945-w)
Supplement: Supplementary file 5 — Additional file 5: Table S3. Characteristics of healthy controls and ALD patients for liver, bile and cholangiocytes. [file 13578_2022_945_MOESM5_ESM.docx]

**Additional file Table S3 Characteristics of healthy controls and ALD patients for liver, bile and cholangiocytes**

| **Groups** | | **Diagnosis** | **Gender** | **Age** | **Sample (Application)** | **Origin** |
| --- | --- | --- | --- | --- | --- | --- |
| Control | 1 | Healthy control liver | Female | 62 | Liver (PCR) | SEKISUI XenoTech |
|  | 2 | Healthy control liver | Female | 24 | Liver (IF, PCR) | SEKISUI XenoTech |
|  | 3 | Healthy control liver | Male | 55 | Liver (PCR) | SEKISUI XenoTech |
|  | 4 | Healthy control liver | Female | 31 | Liver (PCR) | SEKISUI XenoTech |
|  | 5 | Healthy control liver | Female | 58 | Liver (IHC, PCR) | SEKISUI XenoTech |
|  | 6 | Healthy control liver | Female | 56 | Liver (IHC) | SEKISUI XenoTech |
|  | 7 | Healthy control liver | Female | 52 | Liver (IHC) | SEKISUI XenoTech |
|  | 8 | Healthy control liver | Male | 46 | Liver (IF) | SEKISUI XenoTech |
|  | 9 | Healthy control liver | Female | 17 | Liver (IHC, PCR) | SEKISUI XenoTech |
|  | 10 | Healthy control liver | Female | 54 | Bile (Bicarbonate) | IU Health University Hospital |
|  | 11 | Healthy control liver | Male | 55 | Bile (Bicarbonate) | IU Health University Hospital |
|  | 12 | Healthy control liver | Male | 32 | Bile (Bicarbonate) | IU Health University Hospital |
|  | 13 | Healthy control liver | Male | 46 | Liver (IHC, WB, PCR) | IU Health University Hospital |
|  | 14 | Healthy control liver | Male | 29 | Bile (Bicarbonate) | IU Health University Hospital |
|  | 15 | Healthy control liver | Male | 38 | Bile (Bicarbonate) | IU Health University Hospital |
|  | 16 | Healthy control liver | Male | 42 | Bile (Bicarbonate) | IU Health University Hospital |
|  | 17 | Healthy control liver | Male | 54 | Bile (Bicarbonate) | IU Health University Hospital |
|  | 18 | Healthy control liver | Male | 35 | Liver (IHC, WB, PCR) | IU Health University Hospital |
|  | 19 | Healthy control liver | Male | 43 | Liver (IHC, WB, PCR) | IU Health University Hospital |
|  | 20 | Healthy control liver | Male | 25 | Bile (Bicarbonate) | IU Health University Hospital |
|  | 21 | Healthy control liver | Male | 49 | Bile (Bicarbonate) | IU Health University Hospital |
|  | 22 | Healthy control liver | Male | 46 | Liver (IHC, PCR) | IU Health University Hospital |
|  | 23 | Healthy control liver | Male | 29 | Liver (WB) | IU Health University Hospital |
| ALD | 1 | Alcoholic cirrhosis | Female | 67 | Liver (IHC) | IU Health University Hospital |
|  | 2 | Alcoholic cirrhosis | Male | 47 | Liver (IHC, PCR) | IU Health University Hospital |
|  | 3 | Alcoholic cirrhosis | Female | 52 | Liver (IHC, PCR) | IU Health University Hospital |
|  | 4 | Alcoholic cirrhosis | Female | 43 | Liver (IHC) | IU Health University Hospital |
|  | 5 | Alcoholic cirrhosis | Male | 68 | Liver (IHC, PCR) | IU Health University Hospital |
|  | 6 | Alcoholic cirrhosis | Male | 55 | Liver (PCR) | IU Health University Hospital |
|  | 7 | Alcoholic cirrhosis | Male | 33 | Liver (PCR) | IU Health University Hospital |
|  | 8 | Alcoholic cirrhosis | Male | 44 | Liver (PCR) | IU Health University Hospital |
|  | 9 | Alcoholic cirrhosis | Male | 53 | Liver (IHC, WB, PCR) | IU Health University Hospital |
|  | 10 | Alcoholic cirrhosis | Female | 67 | Liver (IHC) | IU Health University Hospital |
|  | 11 | Alcoholic cirrhosis | Female | 60 | Liver (IF, PCR) | IU Health University Hospital |
|  | 12 | Alcoholic cirrhosis | Male | 50 | Liver (IHC, WB, PCR); Bile (Bicarbonate) | IU Health University Hospital |
|  | 13 | Alcoholic cirrhosis | Male | 65 | Liver (PCR) | IU Health University Hospital |
|  | 14 | Alcoholic cirrhosis | Male | 41 | Bile (Bicarbonate) | IU Health University Hospital |
|  | 15 | Alcoholic cirrhosis | Male | 50 | Liver (IF, PCR); Bile (Bicarbonate) | IU Health University Hospital |
|  | 16 | Alcoholic cirrhosis | Female | 64 | Liver (PCR); Bile (Bicarbonate) | IU Health University Hospital |
|  | 17 | Alcoholic cirrhosis | Male | 52 | Liver (PCR) | IU Health University Hospital |
|  | 18 | Alcoholic cirrhosis | Male | 52 | Liver (IF) | IU Health University Hospital |
|  | 19 | Alcoholic cirrhosis | Male | 42 | Liver (PCR) | IU Health University Hospital |
|  | 20 | Alcoholic cirrhosis | Male | 63 | Liver (PCR) | IU Health University Hospital |
|  | 21 | Alcoholic cirrhosis | Male | 43 | Bile (Bicarbonate) | IU Health University Hospital |
|  | 22 | Alcoholic cirrhosis | Male | 36 | Bile (Bicarbonate) | IU Health University Hospital |
|  | 23 | Alcoholic cirrhosis | Male | 51 | Bile (Bicarbonate) | IU Health University Hospital |
|  | 24 | Alcoholic cirrhosis | Male | 62 | Liver (WB); Bile (Bicarbonate) | IU Health University Hospital |
|  | 25 | Alcoholic cirrhosis | Male | 53 | Liver (WB) | IU Health University Hospital |
|  | 26 | Alcoholic cirrhosis | Female | 58 | Bile (Bicarbonate) | IU Health University Hospital |
