# Supplementary material for: Knockout of secretin ameliorates biliary and liver phenotypes during alcohol-induced hepatotoxicity
Source: Cell Biosci. 2023 Jan 9;13:5. doi: 10.1186/s13578-022-00945-w (PMC9830859; doi:10.1186/s13578-022-00945-w)
Supplement: Supplementary file 6 — Additional file 6: Table S4. Characteristics of healthy controls and ALD patients for serum. [file 13578_2022_945_MOESM6_ESM.docx]

**Additional file Table S4 Characteristics of healthy controls and ALD patients for serum**

| **Groups** | | **Diagnosis** | **Gender** | **Age** | **Sample (Application)** | **Origin** |
| --- | --- | --- | --- | --- | --- | --- |
| Control | 1 | Healthy control liver | Male | 40 | Serum (ELISA) | IU Health University Hospital |
|  | 2 | Healthy control liver | Male | 24 | Serum (ELISA) | IU Health University Hospital |
|  | 3 | Healthy control liver | Female | 24 | Serum (ELISA) | IU Health University Hospital |
|  | 4 | Healthy control liver | Female | 23 | Serum (ELISA) | IU Health University Hospital |
|  | 5 | Healthy control liver | Male | 42 | Serum (ELISA) | IU Health University Hospital |
|  | 6 | Healthy control liver | Female | 25 | Serum (ELISA) | IU Health University Hospital |
| ALD | 1 | Alcoholic cirrhosis | Female | 54 | Serum (ELISA) | IU Health University Hospital |
|  | 2 | Alcoholic cirrhosis | Male | 52 | Serum (ELISA) | IU Health University Hospital |
|  | 3 | Alcoholic cirrhosis | Female | 59 | Serum (ELISA) | IU Health University Hospital |
|  | 4 | Alcoholic cirrhosis | Female | 48 | Serum (ELISA) | IU Health University Hospital |
|  | 5 | Alcoholic cirrhosis | Male | 62 | Serum (ELISA) | IU Health University Hospital |
|  | 6 | Alcoholic cirrhosis | Male | 47 | Serum (ELISA) | IU Health University Hospital |
|  | 7 | Alcoholic cirrhosis | Male | 59 | Serum (ELISA) | IU Health University Hospital |
|  | 8 | Alcoholic cirrhosis | Male | 51 | Serum (ELISA) | IU Health University Hospital |
|  | 9 | Alcoholic cirrhosis | Male | 65 | Serum (ELISA) | IU Health University Hospital |
|  | 10 | Alcoholic cirrhosis | Male | 48 | Serum (ELISA) | IU Health University Hospital |
|  | 11 | Alcoholic cirrhosis | Male | 60 | Serum (ELISA) | IU Health University Hospital |
|  | 12 | Alcoholic cirrhosis | Female | 41 | Serum (ELISA) | IU Health University Hospital |
|  | 13 | Alcoholic cirrhosis | Female | 67 | Serum (ELISA) | IU Health University Hospital |
|  | 14 | Alcoholic cirrhosis | Male | 63 | Serum (ELISA) | IU Health University Hospital |
|  | 15 | Alcoholic cirrhosis | Male | 74 | Serum (ELISA) | IU Health University Hospital |
|  | 16 | Alcoholic cirrhosis | Male | 63 | Serum (ELISA) | IU Health University Hospital |
|  | 17 | Alcoholic cirrhosis | Male | 34 | Serum (ELISA) | IU Health University Hospital |
|  | 18 | Alcoholic cirrhosis | Male | 72 | Serum (ELISA) | IU Health University Hospital |
|  | 19 | Alcoholic cirrhosis | Male | 59 | Serum (ELISA) | IU Health University Hospital |
|  | 20 | Alcoholic cirrhosis | Male | 56 | Serum (ELISA) | IU Health University Hospital |
|  | 21 | Alcoholic cirrhosis | Female | 55 | Serum (ELISA) | IU Health University Hospital |
|  | 22 | Alcoholic cirrhosis | Male | 57 | Serum (ELISA) | IU Health University Hospital |
|  | 23 | Alcoholic cirrhosis | Male | 45 | Serum (ELISA) | IU Health University Hospital |
|  | 24 | Alcoholic cirrhosis | Male | 54 | Serum (ELISA) | IU Health University Hospital |
|  | 25 | Alcoholic cirrhosis | Male | 68 | Serum (ELISA) | IU Health University Hospital |
|  | 26 | Alcoholic cirrhosis | Female | 59 | Serum (ELISA) | IU Health University Hospital |
|  | 27 | Alcoholic cirrhosis | Male | 62 | Serum (ELISA) | IU Health University Hospital |
|  | 28 | Alcoholic cirrhosis | Female | 46 | Serum (ELISA) | IU Health University Hospital |
|  | 29 | Alcoholic cirrhosis | Female | 51 | Serum (ELISA) | IU Health University Hospital |
|  | 30 | Alcoholic cirrhosis | Male | 62 | Serum (ELISA) | IU Health University Hospital |
|  | 31 | Alcoholic cirrhosis | Male | 56 | Serum (ELISA) | IU Health University Hospital |
|  | 32 | Alcoholic cirrhosis | Male | 60 | Serum (ELISA) | IU Health University Hospital |
|  | 33 | Alcoholic cirrhosis | Female | 38 | Serum (ELISA) | IU Health University Hospital |
|  | 34 | Alcoholic cirrhosis | Male | 66 | Serum (ELISA) | IU Health University Hospital |
|  | 35 | Alcoholic cirrhosis | Male | 60 | Serum (ELISA) | IU Health University Hospital |
|  | 36 | Alcoholic cirrhosis | Female | 58 | Serum (ELISA) | IU Health University Hospital |
|  | 37 | Alcoholic cirrhosis | Male | 59 | Serum (ELISA) | IU Health University Hospital |
|  | 38 | Alcoholic cirrhosis | Female | 52 | Serum (ELISA) | IU Health University Hospital |
|  | 39 | Alcoholic cirrhosis | Male | 49 | Serum (ELISA) | IU Health University Hospital |
|  | 40 | Alcoholic cirrhosis | Female | 62 | Serum (ELISA) | IU Health University Hospital |
|  | 41 | Alcoholic cirrhosis | Male | 62 | Serum (ELISA) | IU Health University Hospital |
|  | 42 | Alcoholic cirrhosis | Male | 31 | Serum (ELISA) | IU Health University Hospital |
|  | 43 | Alcoholic cirrhosis | Male | 50 | Serum (ELISA) | IU Health University Hospital |
|  | 44 | Alcoholic cirrhosis | Male | 34 | Serum (ELISA) | IU Health University Hospital |
|  | 45 | Alcoholic cirrhosis | Female | 52 | Serum (ELISA) | IU Health University Hospital |
|  | 46 | Alcoholic cirrhosis | Male | 65 | Serum (ELISA) | IU Health University Hospital |
|  | 47 | Alcoholic cirrhosis | Female | 43 | Serum (ELISA) | IU Health University Hospital |
|  | 48 | Alcoholic cirrhosis | Male | 53 | Serum (ELISA) | IU Health University Hospital |
|  | 49 | Alcoholic cirrhosis | Male | 56 | Serum (ELISA) | IU Health University Hospital |
|  | 50 | Alcoholic cirrhosis | Male | 51 | Serum (ELISA) | IU Health University Hospital |

ALD, alcohol-related liver disease.
